# Cross-dehydrogenative coupling of coumarins with Csp^3^–H bonds using an iron–organic framework as a productive heterogeneous catalyst†

**DOI:** 10.1039/c8ra00872h

**Published:** 2018-03-19

**Authors:** Son H. Doan, Vu H. H. Nguyen, Thuong H. Nguyen, Phuc H. Pham, Ngoc N. Nguyen, Anh N. Q. Phan, Thach N. Tu, Nam T. S. Phan

**Affiliations:** Faculty of Chemical Engineering, HCMC University of Technology, VNU-HCM 268 Ly Thuong Kiet, District 10 Ho Chi Minh City Viet Nam ptsnam@hcmut.edu.vn; Center for Innovative Materials and Architectures, VNU-HCM Quarter 6, Linh Trung Ward, Thu Duc District Ho Chi Minh City Viet Nam tnthach@inomar.edu.vn +84 8 38637504 +84 8 38647256 ext. 5681

## Abstract

The iron–organic framework VNU-20 was utilized as an active heterogeneous catalyst for the cross-dehydrogenative coupling of coumarins with Csp^3^–H bonds in alkylbenzenes, cyclohexanes, ethers, and formamides. The combination of DTBP as the oxidant and DABCO as the additive led to high yields of coumarin derivatives. The VNU-20 was more active towards this reaction than numerous other homogeneous and heterogeneous catalysts. Heterogeneous catalysis was confirmed for the cross-dehydrogenative coupling transformation utilizing the VNU-20 catalyst, and the contribution of active iron species in the liquid phase was insignificant. The iron-based framework was reutilized many times for the functionalization of coumarins without a remarkable decline in catalytic efficiency. To the best of our knowledge, these reactions of coumarins have not previously been conducted using heterogeneous catalysts.

## Abstract 2

The iron–organic framework VNU-20 was utilized as an active heterogeneous catalyst for the cross-dehydrogenative coupling of coumarins with Csp^3^–H bonds in alkylbenzenes, cyclohexanes, ethers, and formamides.

## Graphical abstract

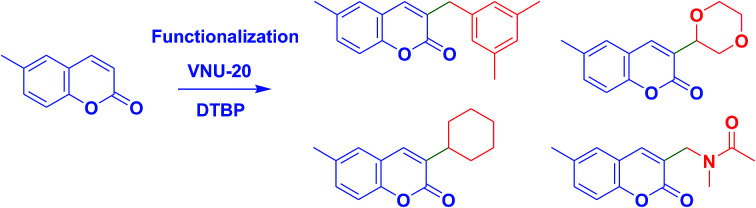

## 1. Introduction

Coumarins represent an important family of precious structural units, largely distributed in a wide range of natural products and pharmaceutical candidates.^1–4^ The functionalization of naturally occurring skeletons has gained significant attention, as interesting and unexpected biological properties would be generated.^5–7^ Among several synthetic strategies, reactions *via* direct C–H bond activation have exhibited significant advantages, avoiding the preparation of prefunctionalized reactants and the purification of intermediate products.^8–10^ However, the transformation of coumarin skeletons *via* direct C–H bond activation has been very limited in the literature. Niu *et al.* previously performed the direct couplings of coumarins with cyclic ethers using a FeCl_3_ catalyst.^11^ Wang *et al.* synthesized a variety of C-3 functionalized coumarins *via* the Cu(OAc)_2_-catalyzed reaction with cyclic ethers and cycloalkanes.^12^ Cao *et al.* developed a novel approach for the direct Csp^2^–H radical trifluoromethylation of coumarins in the presence of Mn(OAc)_3_.^13^ Zhou *et al.* reported the cross-dehydrogenative coupling of coumarins with benzylic Csp^3^–H bonds utilizing Cu(OAc)_2_ catalyst.^14^ Cheng *et al.* prepared several biologically active coumarin derivatives *via* Pd(OAc)_2_-catalyzed intramolecular cross-dehydrogenative coupling reaction.^15^ For more environmentally benign synthetic strategies, transformations of coumarins utilizing heterogeneous catalysts should be explored to achieve simple workup, recyclability, and reusability.

Metal–organic frameworks (MOFs), a significant class of multidimensional crystalline polymeric materials, have been extensively explored during the last decade owing to their encouraging applications in numerous areas.^16–19^ Depending on the nature of metal cations, the structure of organic linkers, as well as synthetic conditions, a broad range of MOFs with various connectivity and symmetry have been generated.^20–23^ Due to the flexibility in designing the active sites on the framework, MOFs have been considered as promising candidates in catalysis field.^20–24^ Both organic and inorganic constituents in MOFs could create catalytically active sites, thus leading to advantages over traditional catalytic materials.^20–23^ Along with MOFs constructed using a single kind of linker, several structures containing a mixture of two or more bridging organic ligands have been explored.^24–26^ If two linkers are present in the frameworks, attractive properties might be achieved.^27^ A variety of organic transformations utilizing iron-based MOFs as heterogeneous catalysts were previously reported in the literature.^28–34^ We recently reported the functionalization of coumarins with *N*,*N*-dimethylanilines in the presence of a mixed-linker iron-based MOF VNU-20 as heterogeneous catalyst.^35^ In this work, we would like to expand the catalytic application of this MOF to the cross-dehydrogenative coupling of coumarins with alkylbenzenes, cycloalkanes, ethers, and formamides. To the best of our knowledge, this functionalization of coumarins was not previously conducted using heterogeneous catalysts.

## 2. Experimental

### 2.1. Synthesis of metal–organic framework VNU-20

In a typical synthesis, 1,3,5-benzenetricarboxylic acid (H_3_BTC; 0.03 g, 0.112 mmol), 2,6-napthalenedicarboxylic acid (H_2_NDC; 0.09 g, 0.42 mmol), and FeCl_2_ (0.09 g, 0.705 mmol) were dissolved in *N*,*N*-dimethylformamide (DMF; 12 mL). The mixture was then sonicated for 5 min to achieve a clear solution. This solution was subsequently divided into glass tubes, which was sealed and placed in an isothermal oven at 200 °C for 72 h. Reddish crystals of VNU-20 were formed during the experiment. Consequently, VNU-20 crystals were washed by DMF (5 × 15 mL) and methanol (5 × 15 mL). The sample was then activated under a dynamic vacuum to obtain activated VNU-20 (0.057 g; 75% yield base on H_3_BTC).

### 2.2. Catalytic studies

In a representative experiment, 6-methylcoumarin (0.040 g, 0.25 mmol), mesitylene (1 mL), 1,4-diazabicyclo[2.2.2]octane (DABCO; 0.028 g, 0.25 mmol), and diphenyl ether (0.04 mL) as internal standard were introduced to a pressurized vial containing the VNU-20 catalyst. Di-*tert*-butylperoxide (DTBP; 0.094 mL, 0.75 mmol) as oxidant was then added dropwise to the vial. The mixture was magnetically stirred at 120 °C for 6 h. The reaction mixture was diluted with ethyl acetate (30 mL). The ethyl acetate solution was washed with HCl solution (5% in water, 3 × 5 mL), and subsequently with saturated NaHCO_3_ solution (3 × 5 mL). The organic layer was dried utilizing anhydrous Na_2_SO_4_. Reaction yields were recorded from GC analysis results concerning the diphenyl ether internal standard. The expected product was isolated using column chromatography. The product structure was confirmed by GC-MS, ^1^H NMR, and ^13^C NMR. For the catalyst recycling studies, the VNU-20 was isolated by centrifugation, washed carefully with DMF and methanol, activated at room temperature under vacuum on a Shlenkline, and reutilized for new catalytic experiments.

## 3. Results and discussion

### 3.1. Catalyst synthesis and characterization

The VNU-20 was synthesized following solvothermal protocol in 75% yield by conducting the reaction between 1,3,5-benzenetricarboxylic acid, 2,6-napthalenedicarboxylic acid, and iron(ii) chloride. The iron-based framework was consequently characterized by utilizing numerous analysis methods (Fig. S1–S7†). Highly sharp peaks existed in the X-ray powder diffraction result, confirming that the VNU-20 was truly crystalline (Fig. S1†). Scanning electron microscopy analysis also supported the crystal form of the iron-based framework (Fig. S2†). Transmission electron microscopy micrograph exhibited a porous structure for the VNU-20 (Fig. S3†). Nevertheless, it was noticeable that the pore structure of the framework was complicated. Nitrogen physisorption measurements demonstrated a pore diameter of less than 10 Å, verifying that the material contained microporous pores (Fig. S4†). Additionally, Langmuir surface areas of 760 m^2^ g^−1^ were obtained for the Fe-MOF, as calculated from isotherm nitrogen physisorption data (Fig. S5†). TGA result revealed that the iron-based framework was stable up to over 300 °C (Fig. S6†). FT-IR spectra of the VNU-20 was also compared to those of 1,3,5-benzenetricarboxylic acid, and 2,6-napthalenedicarboxylic acid (Fig. S7†). These two carboxylic acids have the characterization peak centered at 1710 cm^−1^ and 1674 cm^−1^. Peaks of the coordinated carboxylate group of BTC^3−^ and NDC^2−^ in the VNU-20 were shifted to lower wavelength as higher energy would be needed for the stretching vibration of these functional groups.

### 3.2. Catalytic studies

**Scheme 1:**
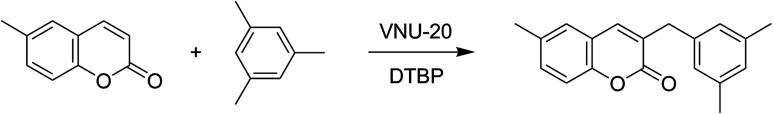
The cross-dehydrogenative coupling of 6-methylcoumarin with mesitylene using the VNU-20 catalyst.

**Fig. 1:**
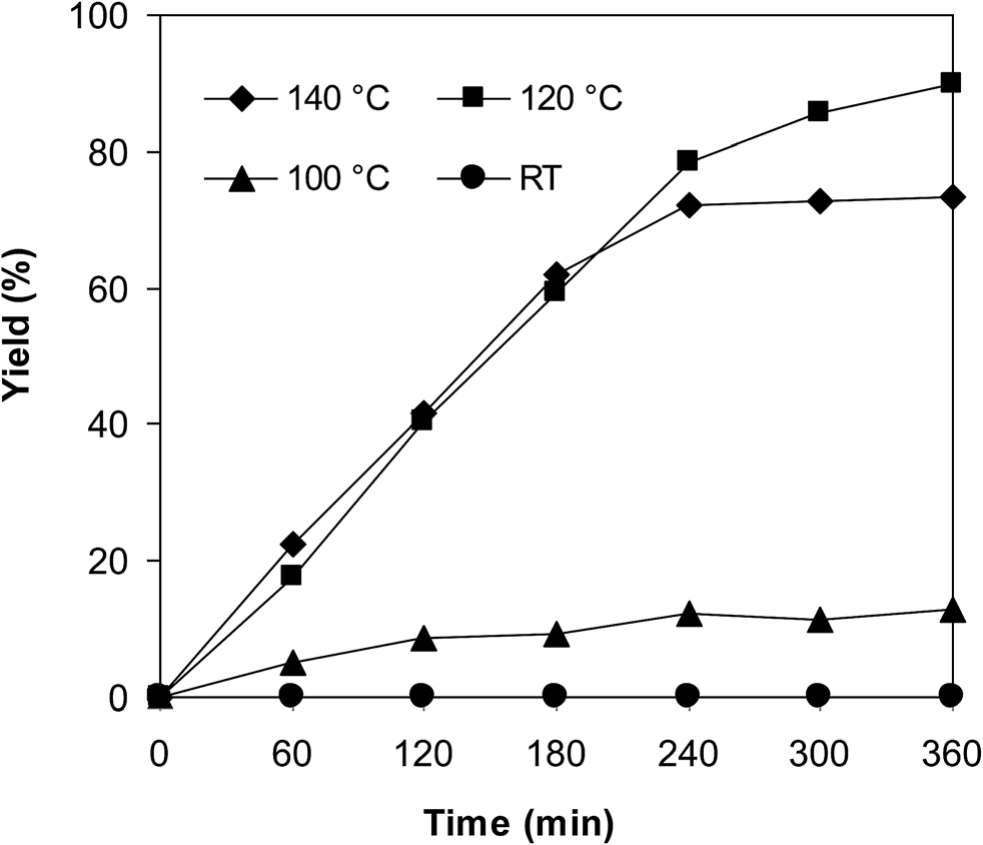
Yields of 3-(3,5-dimethylbenzyl)-6-methyl-2*H*-chromen-2-one *vs.* time at different temperatures.

The iron–organic framework VNU-20 was initially explored as a heterogeneous catalyst for the cross-dehydrogenative coupling of 6-methylcoumarin with mesitylene to produce 3-(3,5-dimethylbenzyl)-6-methyl-2*H*-chromen-2-one as the major product (Scheme 1). First, the influence of temperature on the transformation was studied (Fig. 1). The reaction was conducted at 3 mol% catalyst in mesitylene for 6 h, in the presence of 3 equivalents of DTBP and 1 equivalent of DABCO, at room temperature, 100 °C, 120 °C, and 140 °C, respectively. It was noticed that the cross-dehydrogenative coupling reaction did not occur at ambient temperature, and no evidence of the desired product was detected after 6 h. Boosting the temperature to 100 °C did not accelerate the reaction considerably, affording the major product in only 13% yield. Low yield of the cross-coupled product was still observed for the reaction conducted at 110 °C. It was noticed that the reaction proceeded readily at 120 °C, producing 3-(3,5-dimethylbenzyl)-6-methyl-2*H*-chromen-2-one in 89% yield. However, extending the temperature to 140 °C did not favor the transformation, with 73% yield being recorded after 6 h. This could be explained based on the partial decomposition of coumarin at high temperature.

**Fig. 2:**
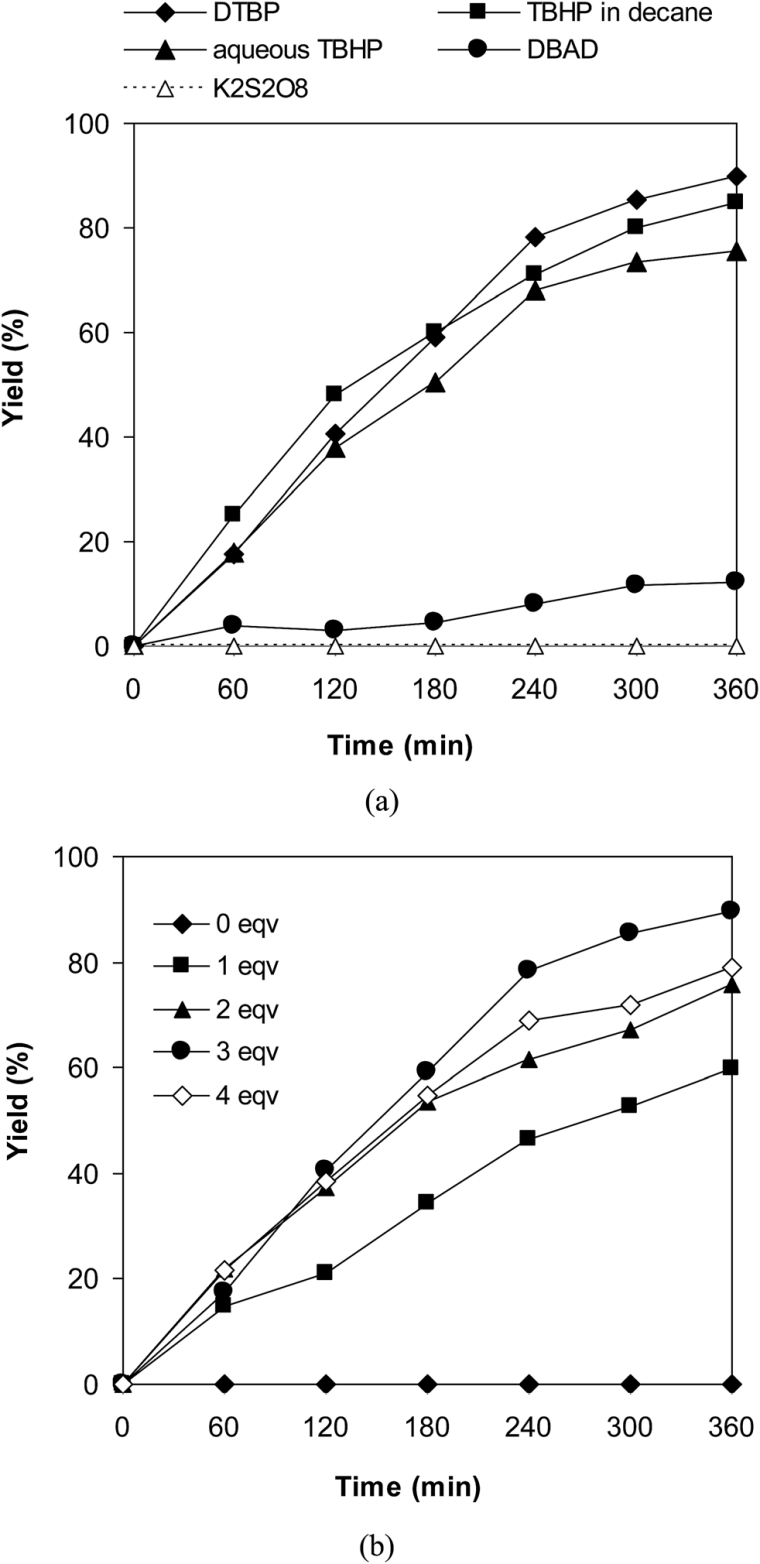
Yields of 3-(3,5-dimethylbenzyl)-6-methyl-2*H*-chromen-2-one *vs.* time with different oxidants (a) and DTBP amounts (b).

Similar to other cross-dehydrogenative coupling reactions, an oxidant should be present in the reaction mixture. We consequently determined to explore the impact of different oxidants on the coupling of 6-methylcoumarin with mesitylene to produce 3-(3,5-dimethylbenzyl)-6-methyl-2*H*-chromen-2-one using the VNU-20 catalyst. The reaction was conducted in mesitylene at 120 °C in the presence of 3 mol% catalyst for 6 h, using 1 equivalent of DABCO, with 3 equivalents of an oxidant, including DTBP, *tert*-butyl hydroperoxide in water (aqueous TBHP), *tert*-butyl hydroperoxide in decane (TBHP in decane) di-*tert*-butyl azodicarboxylate (DBAD), (2,2,6,6-tetramethylpiperidin-1-yl)oxy (TEMPO), hydrogen peroxide (H_2_O_2_), and potassium persulfate (K_2_S_2_O_8_), respectively. Experimental results indicated that DBAD, H_2_O_2_, and K_2_S_2_O_8_ should not be utilized for this reaction, with no trace amounts of product being recorded. Moving to aqueous TBHP, the transformation proceeded to 75% yield after 6 h, while the yield was improved to 84% for the case of TBHP in decane. Compared to these oxidant, DTBP was the oxidant of choice, generating 3-(3,5-dimethylbenzyl)-6-methyl-2*H*-chromen-2-one in 89% yield after 6 h (Fig. 2a). Moreover, the quantity of oxidant also exhibited a considerable influence on the cross-dehydrogenative coupling reaction (Fig. 2b). Best yield was achieved for the reaction utilizing 3 equivalents of DTBP, while increasing the amount of the oxidant resulted in lower yield. It should be noted that no trace evidence of the desired product was detected in the absence of the oxidant.

**Fig. 3:**
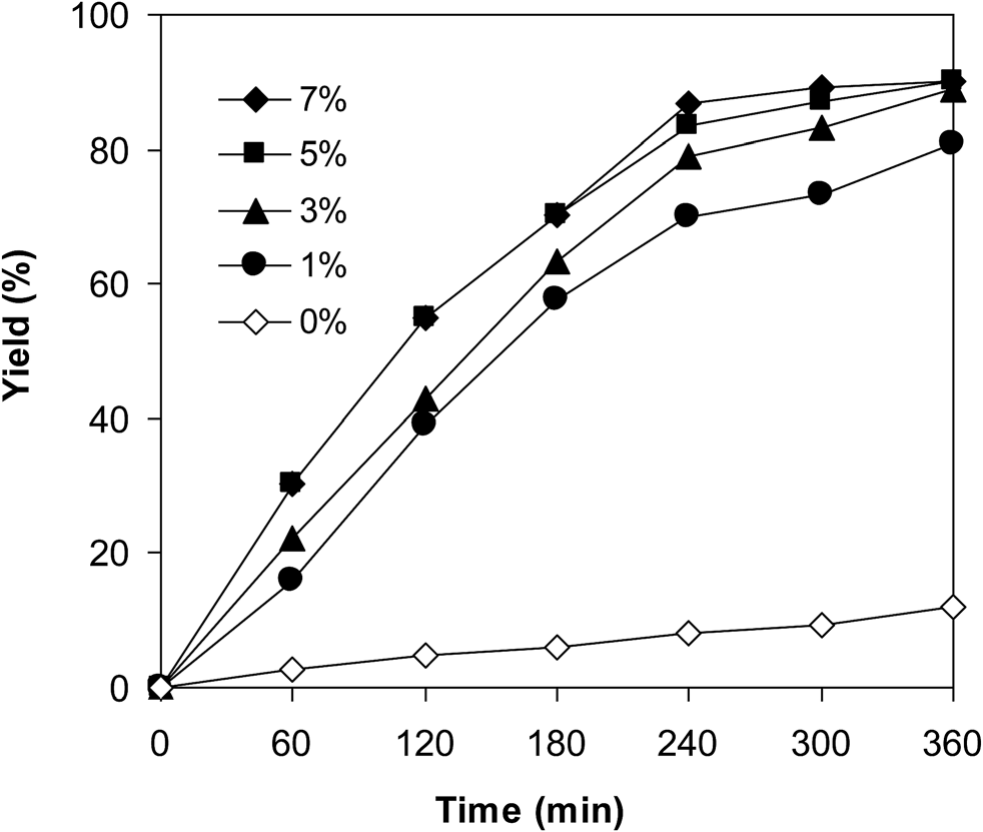
Yields of 3-(3,5-dimethylbenzyl)-6-methyl-2*H*-chromen-2-one *vs.* time at different catalyst amounts.

One more issue that should be investigated for the coupling of 6-methylcoumarin with mesitylene to produce 3-(3,5-dimethylbenzyl)-6-methyl-2*H*-chromen-2-one is the required catalyst amount. Zhou *et al.* previously employed 5 mol% Cu(OAc)_2_ catalyst for the cross-dehydrogenative coupling of coumarins with benzylic Csp^3^–H bonds,^14^ and Niu *et al.* utilized 10 mol% FeCl_3_ catalyst for the direct couplings of coumarins with cyclic ethers.^11^ Wang *et al.* performed the cross-dehydrogenative coupling of coumarins with ethers and cycloalkanes in the presence of 10 mol% Cu(OAc)_2_ catalyst.^12^ The reaction was then performed at 120 °C in mesitylene for 6 h, in the presence of 3 equivalents of DTBP and 1 equivalent of DABCO, at 1 mol%, 3 mol%, 5 mol%, and 7 mol% VNU-20 catalyst, respectively. It was noticed that less than 12% yield of 3-(3,5-dimethylbenzyl)-6-methyl-2*H*-chromen-2-one was detected after 6 h in the absence of the VNU-20, thus indicating that iron species should be required for the transformation. The yield of the major product was remarkably improved in the presence of the iron–organic framework catalyst. Utilizing 1 mol% catalyst, 81% yield was obtained after 6 h. Extending the catalyst amount to 3 mol%, the reaction afforded 89% yield of the desired product after 6 h. Higher initial rates were observed for the reaction utilizing 5 mol% and 7 mol% catalyst. However, after 6 h, 89% yield of 3-(3,5-dimethylbenzyl)-6-methyl-2*H*-chromen-2-one was recorded for both cases (Fig. 3). We therefore employed 3 mol% catalyst for this reaction in further studies.

**Fig. 4:**
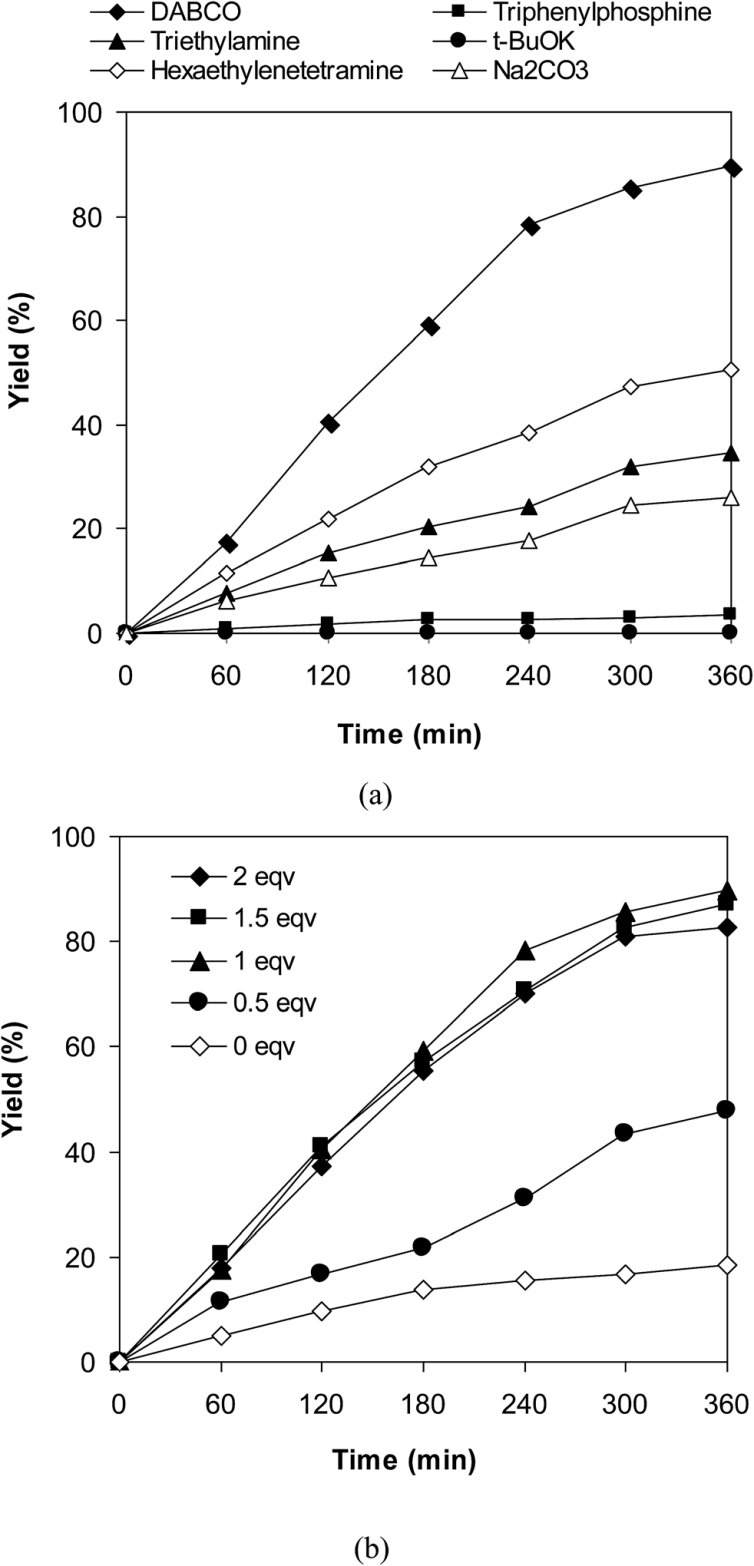
Yields of 3-(3,5-dimethylbenzyl)-6-methyl-2*H*-chromen-2-one *vs.* time with different additives (a) and additive amounts (b).

Experimental results indicated that only 18% yield of 3-(3,5-dimethylbenzyl)-6-methyl-2*H*-chromen-2-one was detected for the reaction in the absence of DABCO as additive. This observation confirmed the importance of the additive in the cross-dehydrogenative coupling reaction using the VNU-20 catalyst. Previously, Niu *et al.* performed the direct couplings of coumarins with cyclic ethers using FeCl_3_ catalyst in the presence of 1 equivalent of DABCO or DBU.^11^ We accordingly explored the transformation with various additives, including DABCO, triphenylphosphine, trimethylamine, hexaethylenetetramine, potassium *tert*-butoxide, and sodium carbonate, respectively (Fig. 4a). The reaction was then conducted at 120 °C in mesitylene for 6 h, in the presence of 3 equivalents of DTBP, with 3 mol% catalyst, using 1 equivalent of the additive. It was noticed that triphenylphosphine, trimethylamine, potassium *tert*-butoxide, and sodium carbonate were not appropriate for the cross-dehydrogenative coupling reaction, affording the expected product in 4%, 35%, 1%, and 26% yields, respectively, after 6 h. Hexaethylenetetramine exhibited better performance, with 51% yield being recorded after 6 h. DABCO emerged as the most suitable additive, producing 3-(3,5-dimethylbenzyl)-6-methyl-2*H*-chromen-2-one in 89% yield after 6 h. Furthermore, it was noticed that the amount of DABCO displayed a considerable impact on the cross-dehydrogenative coupling reaction. Best result was obtained for the reaction using 1 equivalent of DABCO. Increasing or decreasing the amount of DABCO resulted in lower yield of the desired product (Fig. 4b).

**Fig. 5:**
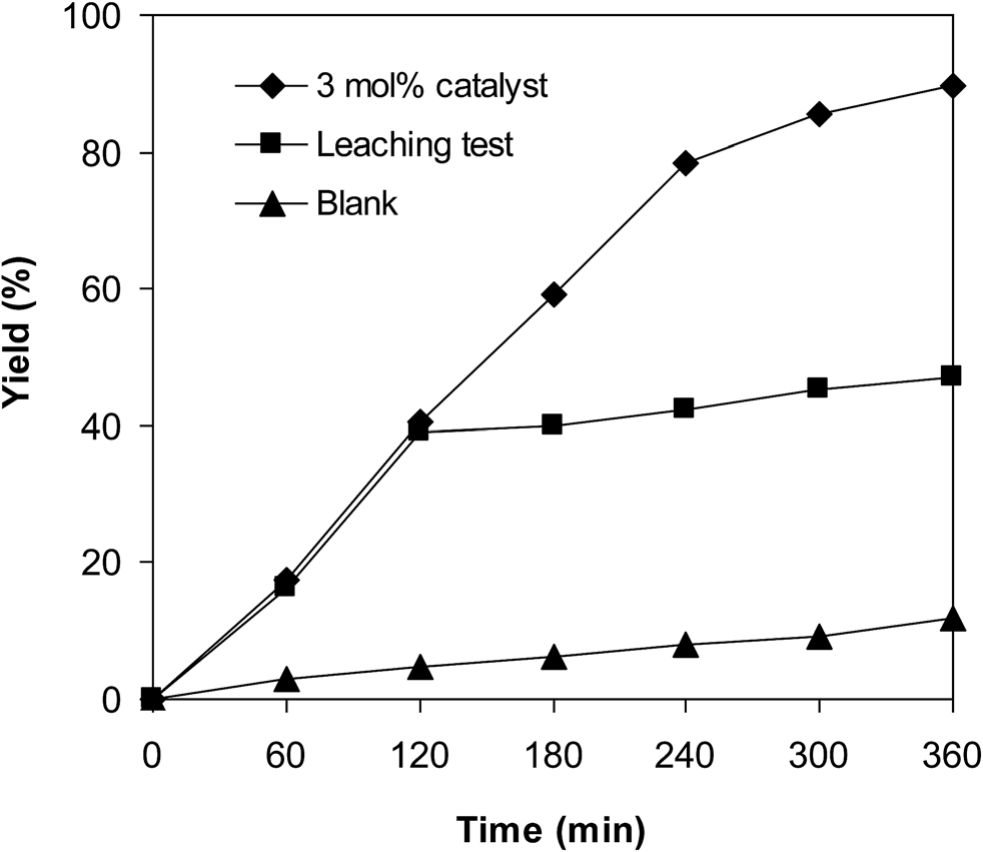
The cross-dehydrogenative coupling reaction was ceased upon the isolation of the VNU-20.

Since the cross-dehydrogenative coupling of 6-methylcoumarin with mesitylene to produce 3-(3,5-dimethylbenzyl)-6-methyl-2*H*-chromen-2-one was performed in solution, it is crucial to explore the leaching phenomenon. Indeed, the formation of product might be achieved *via* homogeneous catalysis because a portion of catalyst was dissolved into the liquid phase. Control experiments were accordingly carried out to confirm if active iron species were migrated from the VNU-20 to mesitylene phase or not. The reaction was performed at 120 °C in mesitylene for 6 h, in the presence of 3 equivalents of DTBP and 1 equivalent of DABCO, at 3 mol% catalyst. After the first 2 h with 39% yield of 3-(3,5-dimethylbenzyl)-6-methyl-2*H*-chromen-2-one being noted, the iron-based framework was isolated. The mesitylene phase was thereafter transferred to a new and clean reactor, and the mixture was heated at 120 °C for additional 4 h. The formation of product during these 4 h, if any, was monitored by GC analysis. Under these conditions, 47% yield of the expected product was recorded after 6 h. It should be noted that the transformation afforded 89% yield in the presence of the VNU-20 after 6 h, and that 12% yield was detected after 6 h in the absence of the catalyst (Fig. 5). These data suggested that the cross-dehydrogenative coupling of 6-methylcoumarin with mesitylene proceeded *via* heterogeneous catalysis, and the contribution of active iron species in liquid phase was insignificant.

**Fig. 6:**
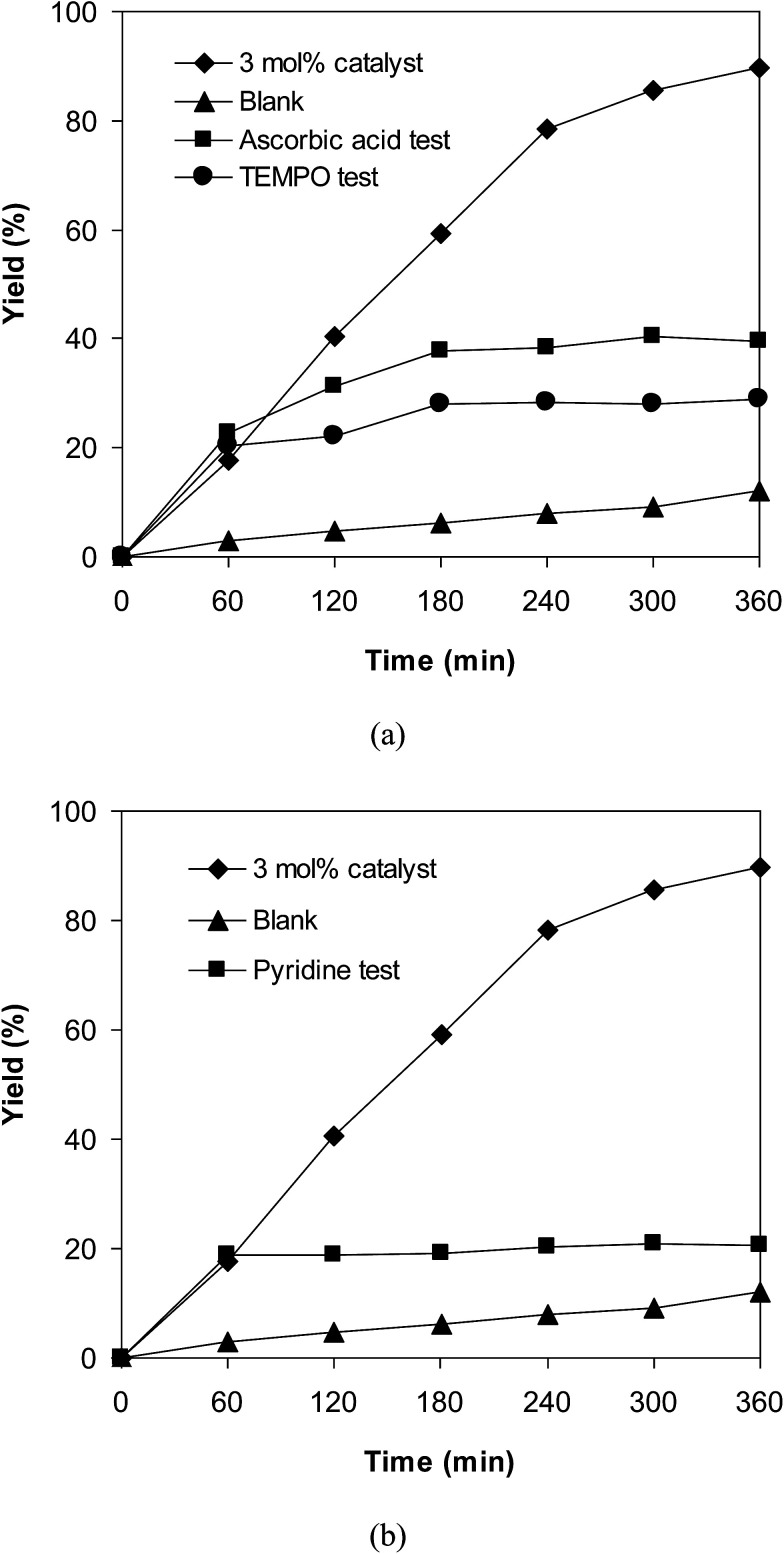
The influence of ascorbic acid and TEMPO as antioxidant (a), and pyridine as catalyst poison (b) on the transformation.

**Scheme 2:**
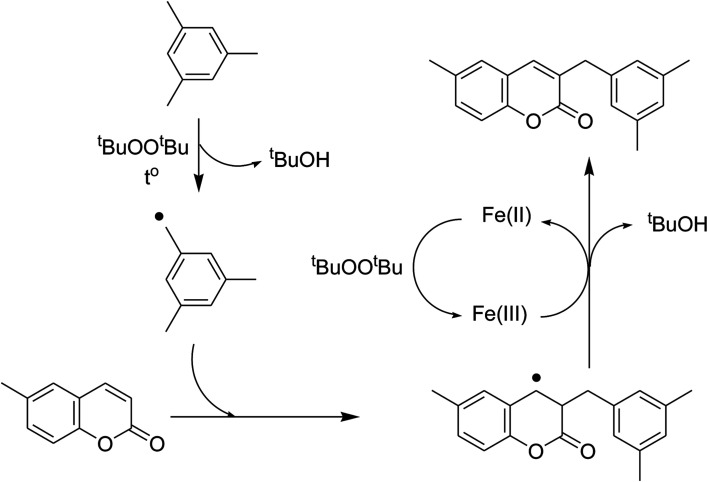
Proposed pathway for the cross-dehydrogenative coupling of 6-methylcoumarin with mesitylene.

To obtain more information about the pathway of the cross-dehydrogenative coupling of 6-methylcoumarin with mesitylene to produce 3-(3,5-dimethylbenzyl)-6-methyl-2*H*-chromen-2-one, additional control experiments were also executed. First, the reaction was performed at 120 °C in mesitylene for 6 h, in the presence of 3 equivalents of DTBP and 1 equivalent of DABCO, at 3 mol% catalyst. After 1 h reaction period, ascorbic acid as antioxidant was introduced to the reactor, and the mixture was heated at 120 °C for additional 5 h. The presence of ascorbic acid in the reaction mixture displayed a remarkable influence on the formation of 3-(3,5-dimethylbenzyl)-6-methyl-2*H*-chromen-2-one. Certainly, this experiment led to 39% yield of the desired product after 6 h reaction time. Similarly, (2,2,6,6-tetramethylpiperidin-1-yl)oxyl (TEMPO) as antioxidant was employed, and only 29% yield was detected after 6 h (Fig. 6a). It should be noted that the reaction afforded 89% yield in the presence of the VNU-20 after 6 h. These data implied that ascorbic acid or TEMPO trapped the radicals generated in the cycle of the catalytic conversion, therefore ceasing the cross-dehydrogenative coupling reaction. In an other test, pyridine as a catalyst poison, was used to deactivate the catalyst after the first 1 h reaction period, and the mixture was heated at 120 °C for additional 5 h. It was noted that the presence of pyridine considerably refrained the transformation (Fig. 6b). The low yield of 3-(3,5-dimethylbenzyl)-6-methyl-2*H*-chromen-2-one could be explained based on the strong interaction between Lewis acid sites on the VNU-20 and the pyridine as a Lewis base. Indeed, Dhakshinamoorthy *et al.* previously pointed out that the interaction of pyridine as a base with the Lewis acid sites in metal–organic frameworks resulted in the deactivation of the MOF-based catalysts.^36^ Therefore, the free coordination iron sites in the VNU-20 framework should be responsible for the catalytic coupling reaction, and the deactivation of these sites would terminate the transformation. From these observations and previous reports,^7,8,10^ a plausible mechanism was suggested (Scheme 2). Initially, hydrogen extraction from mesitylene by DTBP created a stable benzylic radial. Next, the interaction between 6-methylcoumarin and this radical produced another benzylic radical. Releasing of a proton, the coupling product was formed, and the Fe(ii) species was regenerated.

**Fig. 7:**
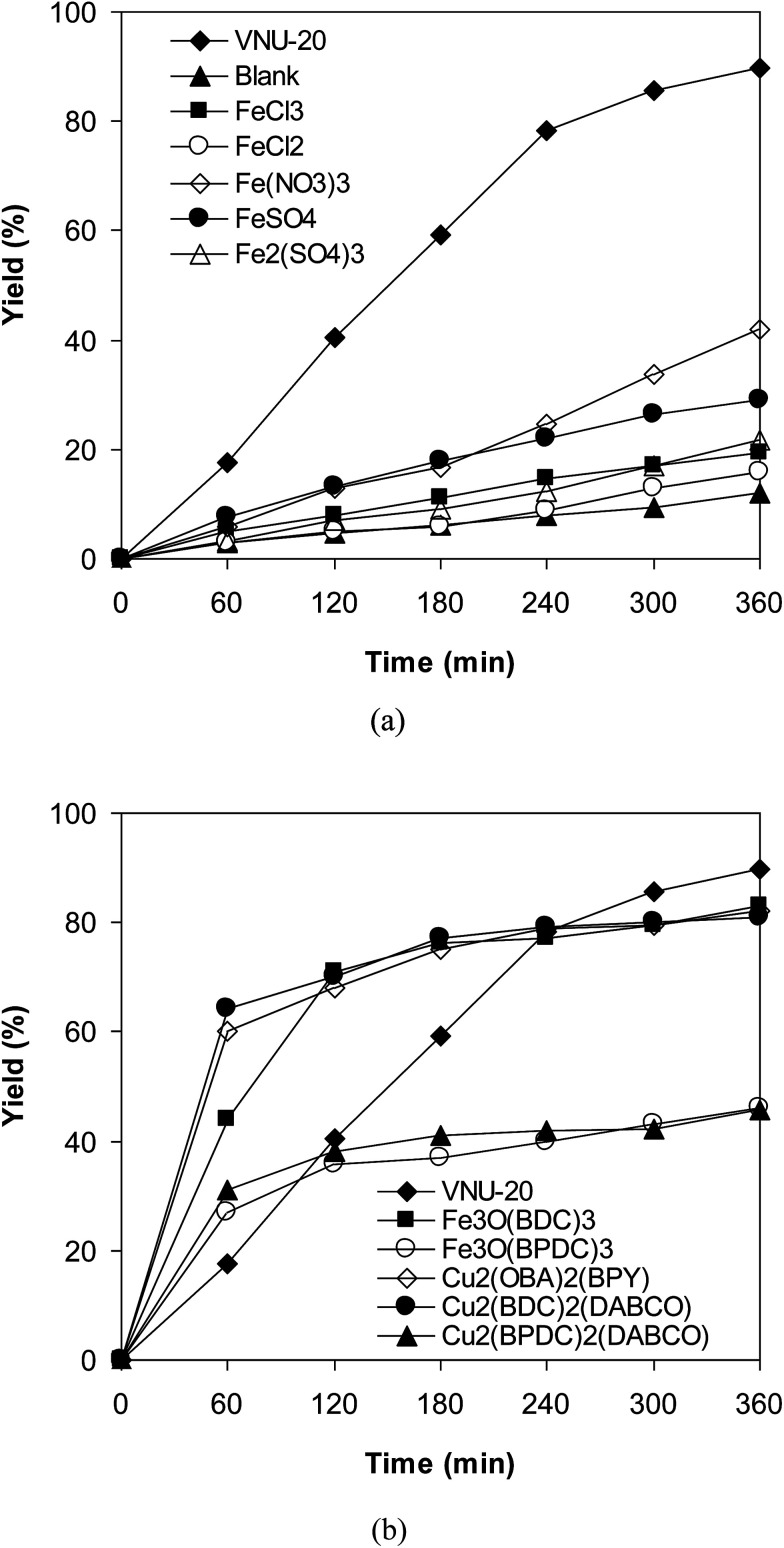
Yields of 3-(3,5-dimethylbenzyl)-6-methyl-2*H*-chromen-2-one *vs.* time with different homogeneous (a) and heterogeneous (b) catalysts.

To emphasize the remarkable aspect of this iron–organic framework, the catalytic activity of the VNU-20 in the cross-dehydrogenative coupling of 6-methylcoumarin with mesitylene to produce 3-(3,5-dimethylbenzyl)-6-methyl-2*H*-chromen-2-one was compared to a series of homogeneous catalysts and heterogeneous catalysts. The reaction was performed at 120 °C in mesitylene for 6 h, in the presence of 3 equivalents of DTBP and 1 equivalent of DABCO, at 3 mol% catalyst. The transformation progressed slowly in the presence of FeCl_2_ catalyst, generating the desired product in only 16% yield after 6 h. FeCl_3_ displayed similar activity, with 19% yield being noted after 6 h under similar conditions. FeSO_4_ and Fe_2_(SO_4_)_3_ were also not appropriate as catalysts for this reaction, forming 3-(3,5-dimethylbenzyl)-6-methyl-2*H*-chromen-2-one in 29% and 22% yields, respectively, after 6 h. Fe(NO_3_)_3_ was more active than other iron salts, and using this catalyst resulted in 42% yield after 6 h. Interestingly, the VNU-20 offered remarkably higher catalytic activity towards the cross-dehydrogenative coupling than these homogeneous catalysts, with 89% yield being recorded after 6 h (Fig. 7a).

Next, several MOF-based catalysts were explored for this reaction, including VNU-20, Fe_3_O(BDC)_3_ [BDC = 1,4-benzenedicarboxylate], Fe_3_O(BPDC)_3_ [BPDC = 4,4′-biphenyldicarboxylate], Cu_2_(OBA)_2_(BPY) [OBA = 4,4′-oxybis(benzoate); BPY = 4,4′-bipyridine], Cu_2_(BDC)_2_(DABCO), and Cu_2_(BPDC)_2_(DABCO). Among these solid catalysts, Fe_3_O(BPDC)_3_ and Cu_2_(BPDC)_2_(DABCO) expressed low catalytic efficiency, though 46% yield of the expected product was observed after 6 h. Utilizing Fe_3_O(BDC)_3_, Cu_2_(OBA)_2_(BPY), Cu_2_(BDC)_2_(DABCO) as catalysts led to higher initial rates. However, 81% and 82% yields were obtained for the reaction after 6 h. The transformation using the VNU-20 catalyst proceeded with lower initial rate in the first 4 h reaction time. Nevertheless, the yield of 3-(3,5-dimethylbenzyl)-6-methyl-2*H*-chromen-2-one was upgraded to 89% after 6 h (Fig. 7b). Previously, Zhou *et al.* performed the cross-dehydrogenative coupling of coumarins with benzylic Csp^3^–H bonds at 100 °C for 24 h utilizing 5 mol% Cu(OAc)_2_ catalyst.^14^ Wang *et al.* conducted the coupling reaction of coumarins with cyclic ethers and cycloalkanes at 100 °C for 24 h in the presence of 10 mol% Cu(OAc)_2_ catalyst.^12^ Niu *et al.* carried out similar transformation at 120 °C for 36 h using 10 mol% FeCl_3_ catalyst.^11^ Although interesting results were achieved, it was difficult to recycle and reuse these homogeneous catalysts. The fact that coumarin derivatives were produced by utilizing a recyclable catalyst was therefore of significant advantages.

**Fig. 8:**
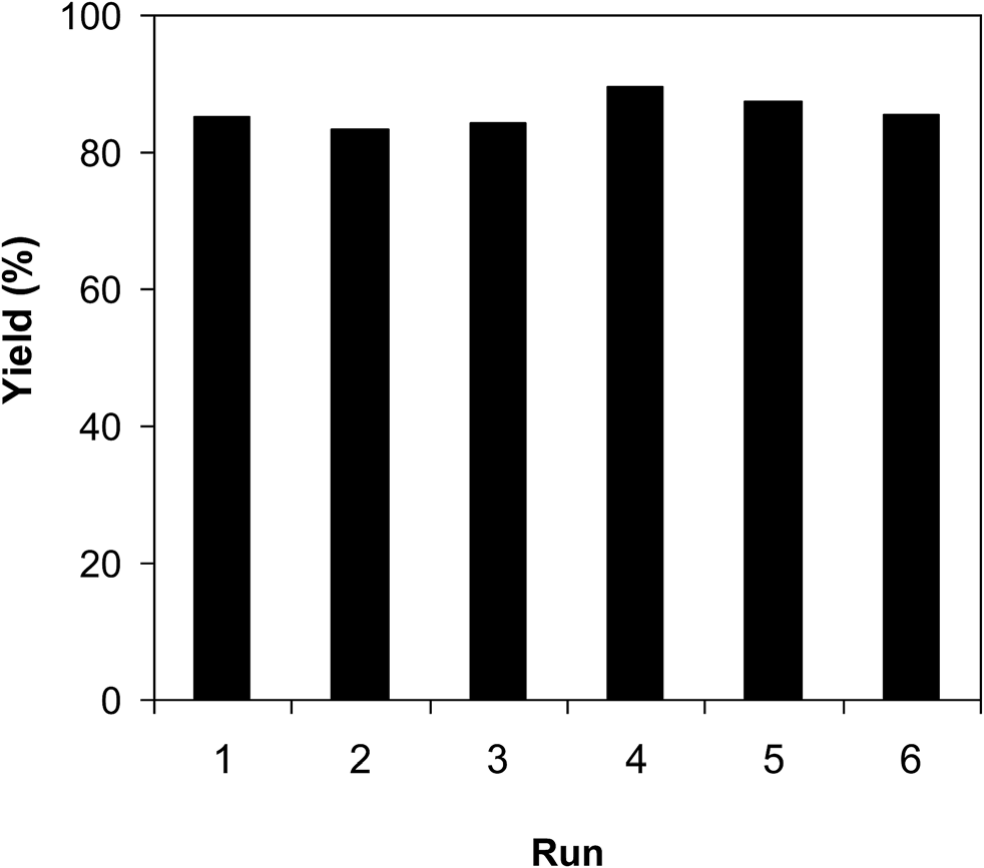
Catalyst reutilization studies.

As pointed out previously, compared to numerous homogeneous and heterogeneous catalysts, the VNU-20 was more active towards the cross-dehydrogenative coupling of 6-methylcoumarin with mesitylene to produce 3-(3,5-dimethylbenzyl)-6-methyl-2*H*-chromen-2-one. To spotlight the remarkable aspect of using this iron-based MOF in this transformation, the promptness of reusability was accordingly explored with 6 sequential catalytic runs. The reaction was performed at 120 °C in mesitylene for 6 h, in the presence of 3 equivalents of DTBP and 1 equivalent of DABCO, at 3 mol% catalyst. After each experiment, the VNU-20 was isolated by centrifugation, washed carefully with DMF and methanol, and activated at room temperature under vacuum on a Shlenkline. The recovered VNU-20 was subsequently reutilized for new catalytic experiments using similar reaction conditions. GC analysis results indicated that it was possible to reutilize the VNU-20 many times for the formation of 3-(3,5-dimethylbenzyl)-6-methyl-2*H*-chromen-2-one without a remarkable decline in catalytic efficiency. Certainly, the cross-dehydrogenative coupling reaction afforded 85% yield in the 6th catalytic run (Fig. 8). Additionally, some characterization experiments were conducted to explore if the structure of the VNU-20 was maintained. FT-IR results of the recovered framework (Fig. S32†) was similar to those of the fresh catalyst. The crystallinity of the VNU-20 was not destroyed during the catalytic experiments, although slight difference was noted in XRD analysis results (Fig. S33†).

The research scope was consequently extended to the cross-dehydrogenative coupling of coumarin or 6-methylcoumarin with different benzylic Csp^3^–H bonds (Table 1). The reaction was performed at 120 °C in mesitylene for 6 h, in the presence of 3 equivalents of DTBP and 1 equivalent of DABCO, at 3 mol% catalyst. The products were purified on silica gel by column chromatography. The reaction between coumarin and 1-bromo-4-methylbenzene afforded 3-(4-bromobenzyl)-2*H*-chromen-2-one (entry 1, Table 1) in 72% yield. Moving to *p*-xylene, the coupling reaction with coumarin proceeded to 76% yield of 3-(4-methylbenzyl)-2*H*-chromen-2-one (entry 2, Table 1). Similarly, 3-(3-methylbenzyl)-2*H*-chromen-2-one (entry 3, Table 1) was obtained in 80% yield for the case of *m*-xylene. *o*-Xylene was noticed to be slightly less reactive towards the coupling reaction with coumarin, though 3-(2-methylbenzyl)-2*H*-chromen-2-one (entry 4, Table 1) was generated in 67% yield. Using this protocol, the reaction between coumarin and mesitylene produced 3-(3,5-dimethylbenzyl)-2*H*-chromen-2-one (entry 5, Table 1) in 88% yield, while 87% yield of 3-(3,5-dimethylbenzyl)-6-methyl-2*H*-chromen-2-one (entry 6, Table 1) was achieved for the case of 6-methylcoumarin. Next, cyclohexane was employed for the coupling with 6-methylcoumarin, and 3-cyclohexyl-6-methyl-2*H*-chromen-2-one (entry 7, Table 1) was produced in 60% yield. The work was then expanded to the reaction with cyclic ethers using the VNU-20 catalyst. 1,4-Dioxane was noticed to be reactive in this reaction, forming 3-(1,4-dioxan-2-yl)-2*H*-chromen-2-one (entry 8, Table 1) in 78% yield. Similarly, tetrahydro-2*H*-pyran and tetrahydrofuran could be utilized as reactants for this reaction. Under the same conditions, 3-(tetrahydro-2*H*-pyran-2-yl)-2*H*-chromen-2-one (entry 9, Table 1), and 3-(tetrahydrofuran-2-yl)-2*H*-chromen-2-one (entry 10, Table 1) were obtained in 74% and 73% yields, respectively. Interestingly, DMAc was also found to be reactive towards this reaction, producing *N*-methyl-*N*-((2-oxo-2*H*-chromen-3-yl)methyl)acetamide (entry 11, Table 1) and *N*-methyl-*N*-((6-methyl-2-oxo-2*H*-chromen-3-yl)methyl)acetamide (entry 12, Table 1) in 97% and 90% yields, respectively.

**Table 1:** The cross-dehydrogenative coupling of coumarins utilizing the VNU-20 catalyst

Entry	Reactant 1	Reactant 2	Product	Isolated yields (%)
1	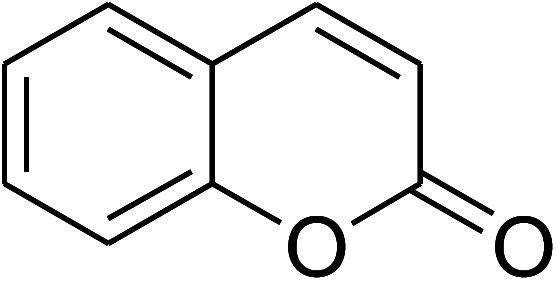	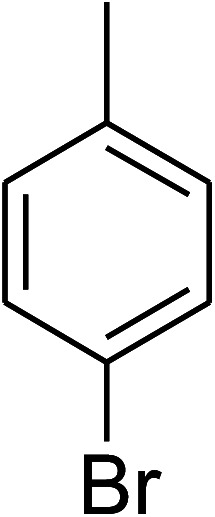	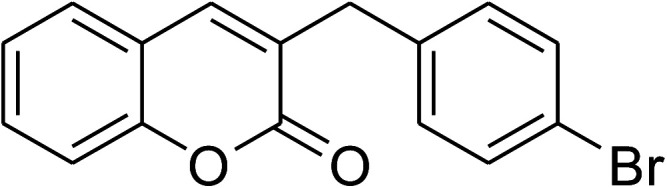	72
2	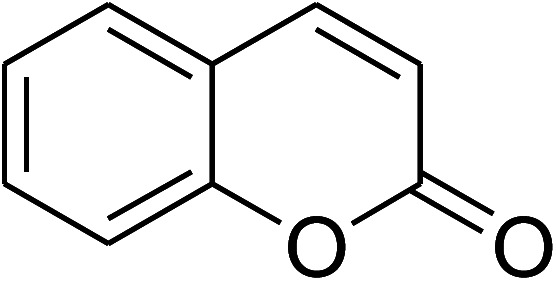	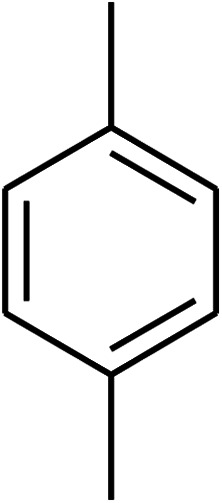	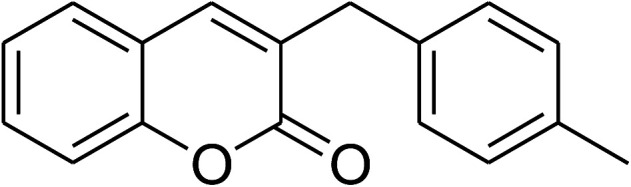	76
3	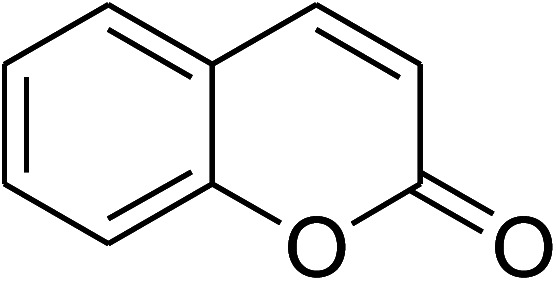	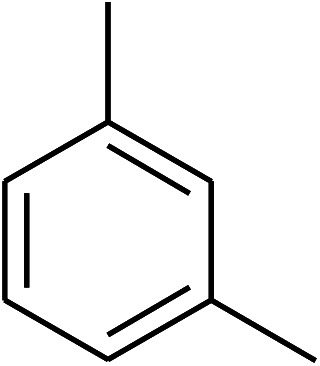	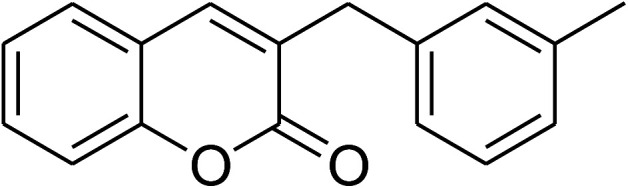	80
4	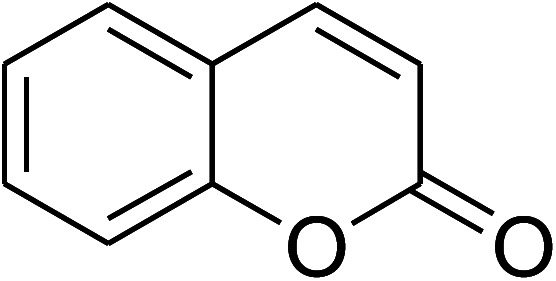	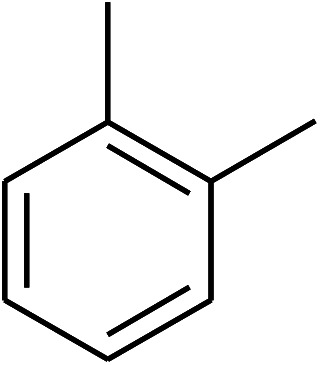	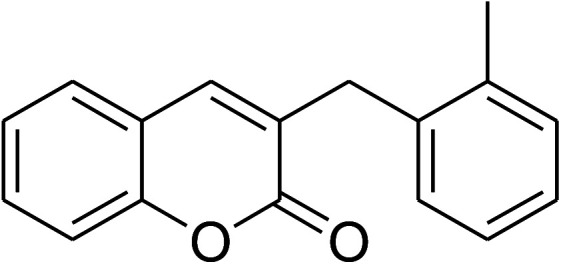	67
5	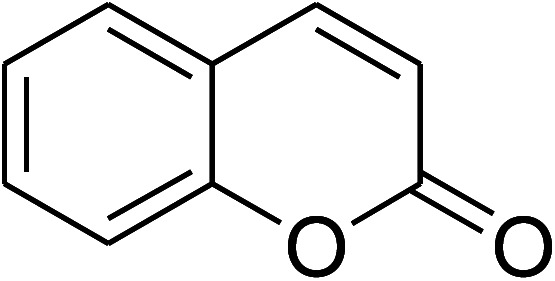	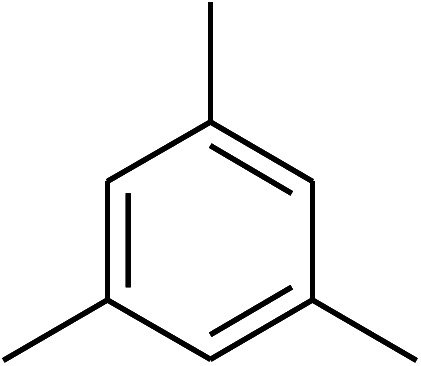	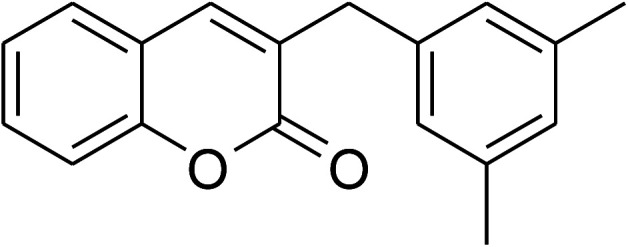	88
6	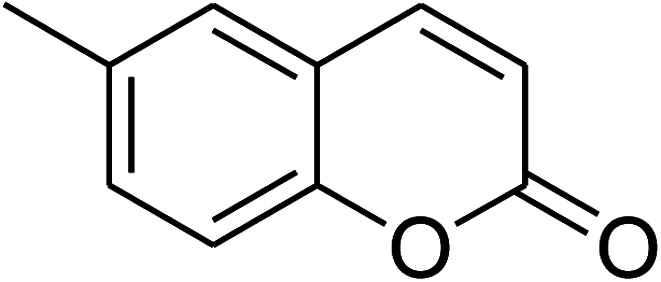	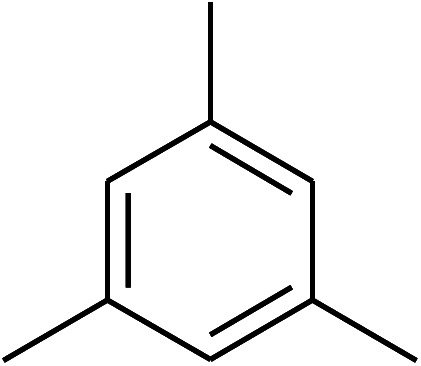	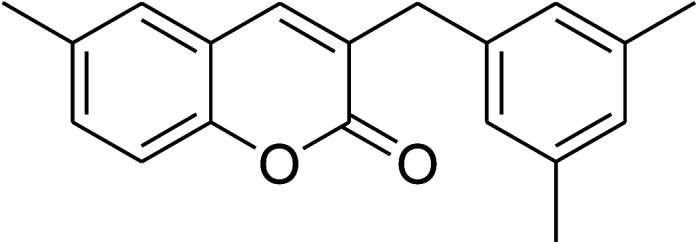	87
7	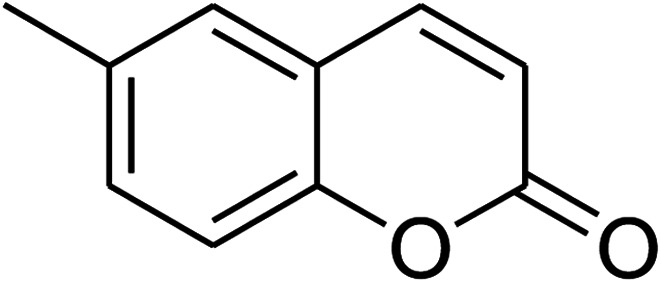	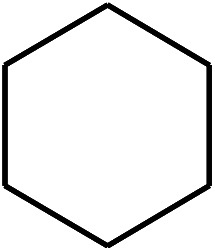	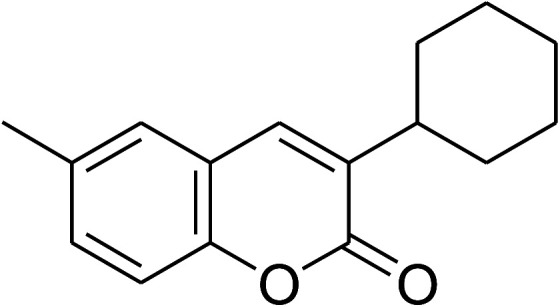	60
8	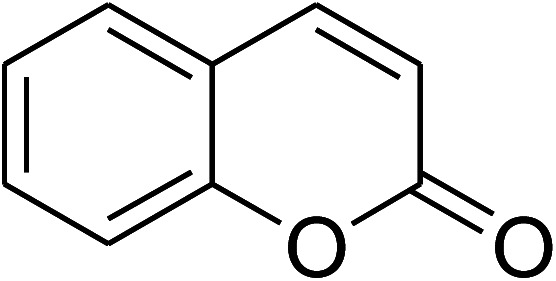	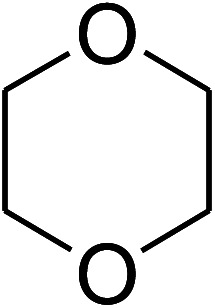	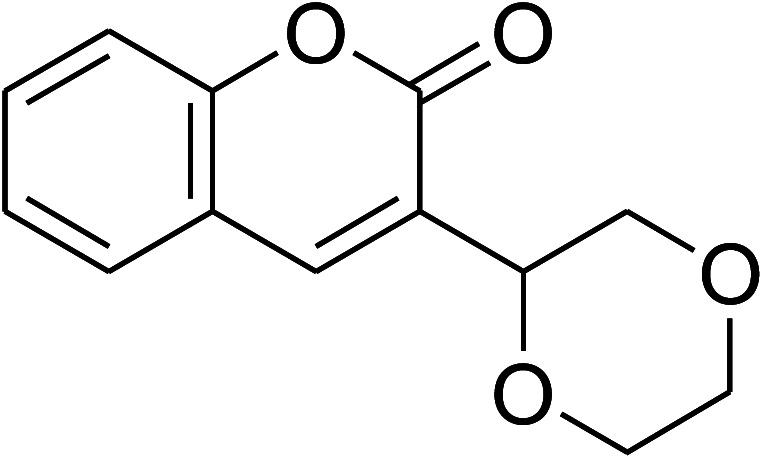	78
9	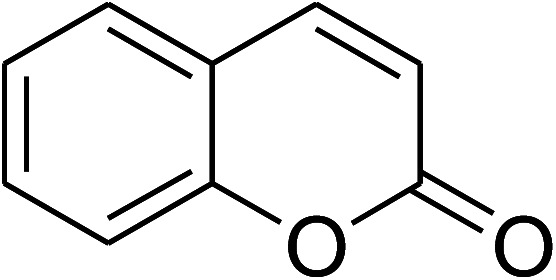	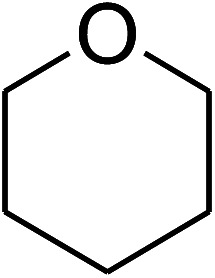	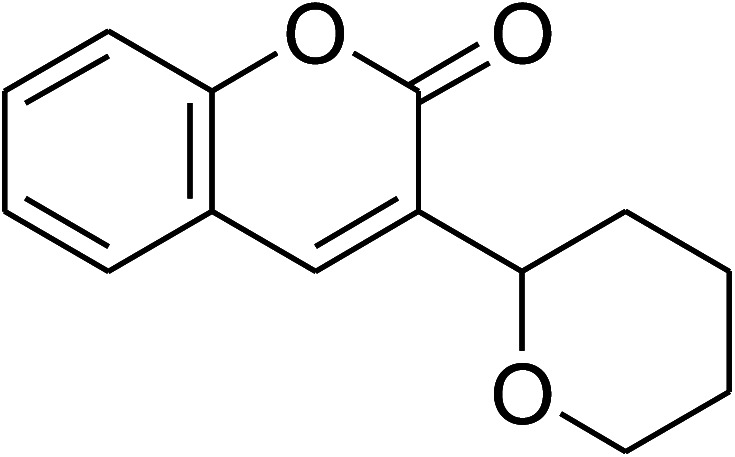	74
10	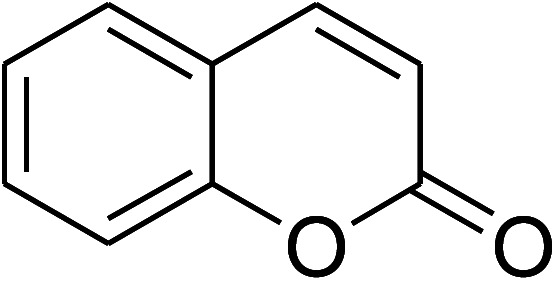	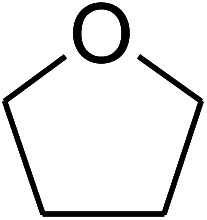	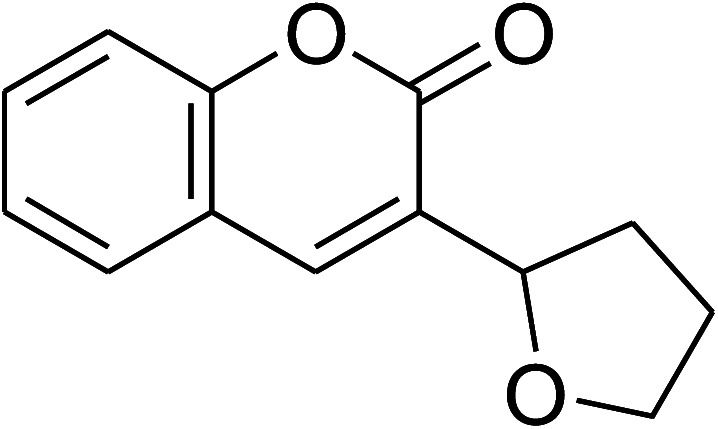	73
11	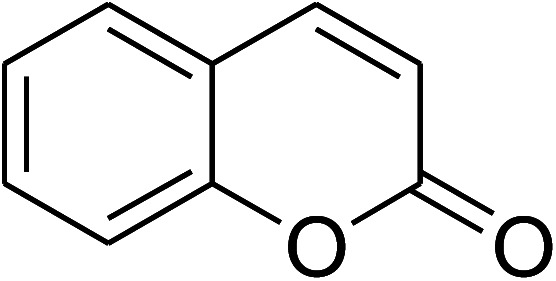	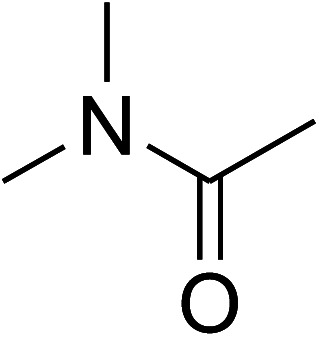	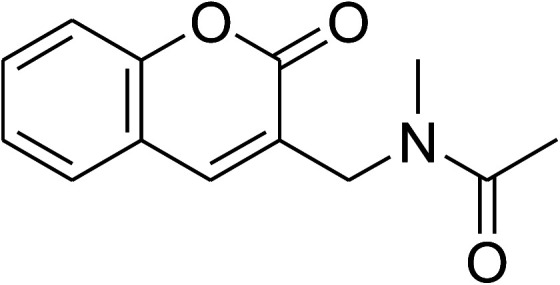	97
12	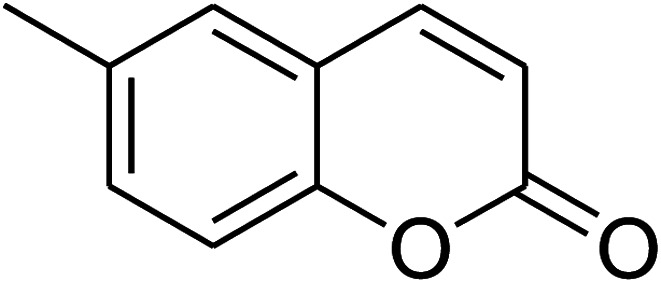	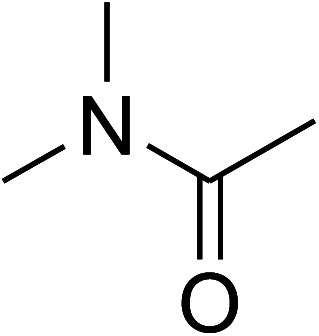	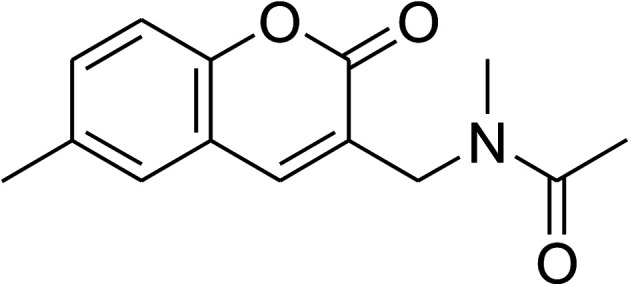	90

## 4. Conclusions

Iron–organic framework VNU-20 emerged as an active heterogeneous catalyst for the cross-dehydrogenative coupling of coumarins with different benzylic Csp^3^–H bonds. The reaction required an oxidant, and a basic additive. The combination of DTBP as the oxidant and DABCO as the additive led to high yields of coumarin derivatives. The VNU-20 was more active towards this transformation than a series of homogeneous and heterogeneous catalysts, thus emphasizing the significant aspect of utilizing this iron–organic framework for the reaction. Heterogeneous catalysis was confirmed for the cross-dehydrogenative coupling transformation utilizing the VNU-20 catalyst, and the contribution of active iron species in liquid phase was insignificant. It was possible to reutilize the VNU-20 many times for the formation of 3-(3,5-dimethylbenzyl)-6-methyl-2*H*-chromen-2-one without a remarkable decline in catalytic efficiency. Moreover, the protocol was also expanded to the cross-dehydrogenative coupling of coumarins with cycloalkanes, ethers, and formamides. The fact that coumarins could be functionalized *via* cross-dehydrogenative coupling with Csp^3^–H bonds in the presence of a recyclable heterogeneous catalyst would be profitable to the chemical and pharmaceutical industry.

## Conflicts of interest

There are no conflicts to declare.

## Supplementary Material

RA-008-C8RA00872H-s001
