# Supplementary material for: Cross-dehydrogenative coupling of coumarins with Csp3–H bonds using an iron–organic framework as a productive heterogeneous catalyst
Source: RSC Adv. 2018 Mar 19;8(20):10736–45. doi: 10.1039/c8ra00872h (PMC9078982; doi:10.1039/c8ra00872h)
Supplement: RA-008-C8RA00872H-s001 [file RA-008-C8RA00872H-s001.pdf]

Electronic Supplementary Material (ESI) for RSC Advances.
This journal is © The Royal Society of Chemistry 2018

Cross-dehydrogenative coupling of coumarins with Csp^3-H bonds using an iron-organic framework as a productive heterogeneous catalyst

Son H. Doan[a], Vu H. H. Nguyen[a], Thuong H. Nguyen[a], Phuc H. Pham[a], Ngoc N. Nguyen[b], Anh N. Q. Phan[a], Thach N. Tu[b*], Nam T. S. Phan[a*]

[a]Faculty of Chemical Engineering, HCMC University of Technology, VNU-HCM, 268 Ly Thuong Kiet, District 10, Ho Chi Minh City, Viet Nam

[b]Center for Innovative Materials and Architectures, VNU-HCM, Quarter 6, Linh Trung Ward, Thu Duc District, Ho Chi Minh City , Viet Nam

*Email: ptsnam@hcmut.edu.vn, tnthach@inomar.edu.vn

Ph: (+84 8) 38647256 ext. 5681 Fx: (+84 8) 38637504

Supporting Information

Materials and instrumentation

All reagents and starting materials were obtained commercially from Sigma-Aldrich and Merck, and were used as received without any further purification unless otherwise noted. Nitrogen physisorption measurements were conducted using a Micromeritics 2020 volumetric adsorption analyzer system. Samples were pretreated by heating under vacuum at 150 ^oC for 3 h. A Netzsch Thermoanalyzer STA 409 was used for thermogravimetric analysis (TGA) with a heating rate of 10 ^oC/min under a nitrogen atmosphere. X-ray powder diffraction (XRD) patterns were recorded using a Cu Kα radiation source on a D8 Advance Bruker powder diffractometer. Scanning electron

microscopy studies were conducted on a S4800 Scanning Electron Microscope (SEM). Transmission electron microscopy studies were performed using a JEOL JEM 1400 Transmission Electron Microscope (TEM) at 80 kV. The sample was dispersed on holey carbon grids for TEM observation. Elemental analysis with atomic absorption spectrophotometry (AAS) was performed on an AA-6800 Shimadzu. Fourier transform infrared (FT-IR) spectra were obtained on a Nicolet 6700 instrument, with samples being dispersed on potassium bromide pallets.

Gas chromatographic (GC) analyses were performed using a Shimadzu GC 2010-Plus equipped with a flame ionization detector (FID) and an SPB-5 column (length = 30 m, inner diameter = 0.25 mm, and film thickness = 0.25 μm). The temperature program for GC analysis held samples at 120 ^{o}C for 0.5 min; heated them from 120 to 130 ^{o}C at 40 ^{o}C/min; held them at 130 ^{o}C for 1 min; heated them from 130 to 280 ^{o}C at 40 ^{o}C/min; and finally held them at 280 ^{o}C for 1.5 min. Inlet and detector temperatures were set constant at 280 ^{o}C. The GC yield was calculated using diphenyl ether as the internal standard. GC-MS analyses were analyzed on a Shimadzu GCMS-QP2010Ultra with a ZB-5MS column (length = 30 m, inner diameter = 0.25 mm, and film thickness = 0.25 μm). The temperature program for GC-MS analysis held samples at 50 ^{o}C for 2 min; heated samples from 50 to 280°C at 10 ^{o}C/min and held them at 280 ^{o}C for 10 min. Inlet temperature was set constant at 280 ^{o}C. MS spectra were compared with the spectra gathered in the NIST library. The ^{1}H NMR and ^{13}C NMR were recorded on Bruker AV 500 spectrometers using residual solvent peak as a reference.

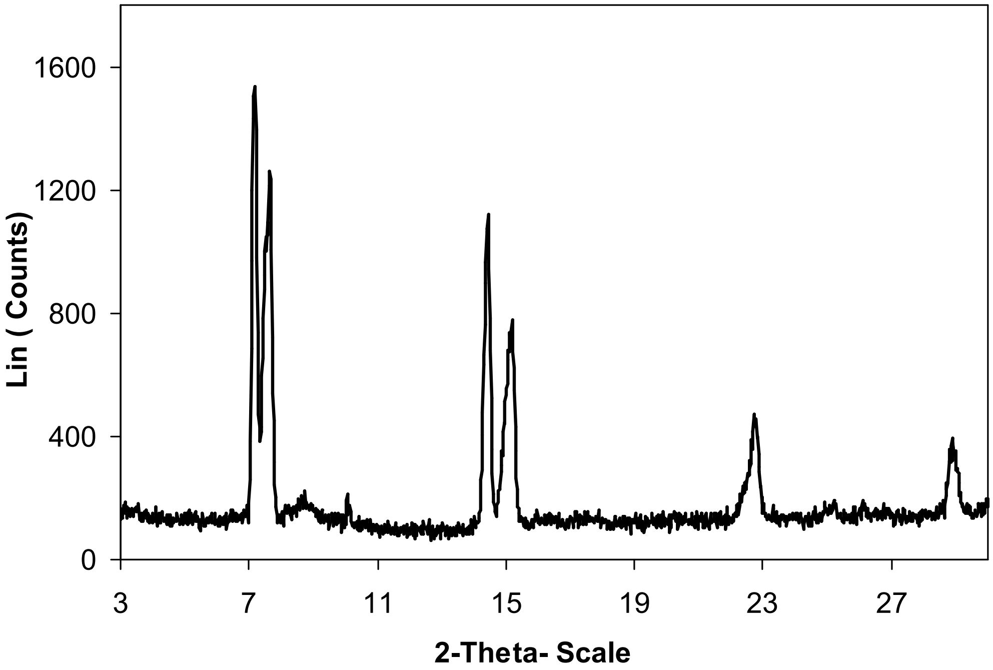

Fig. S1. X-ray powder diffractograms of the VNU-20.

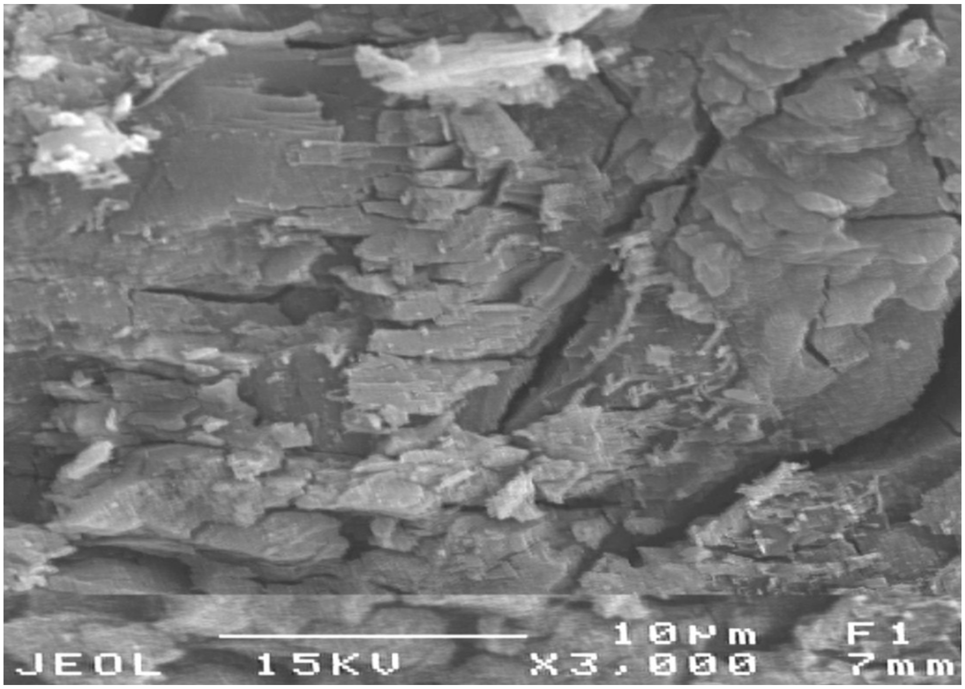

Fig. S2. SEM micrograph of the VNU-20.

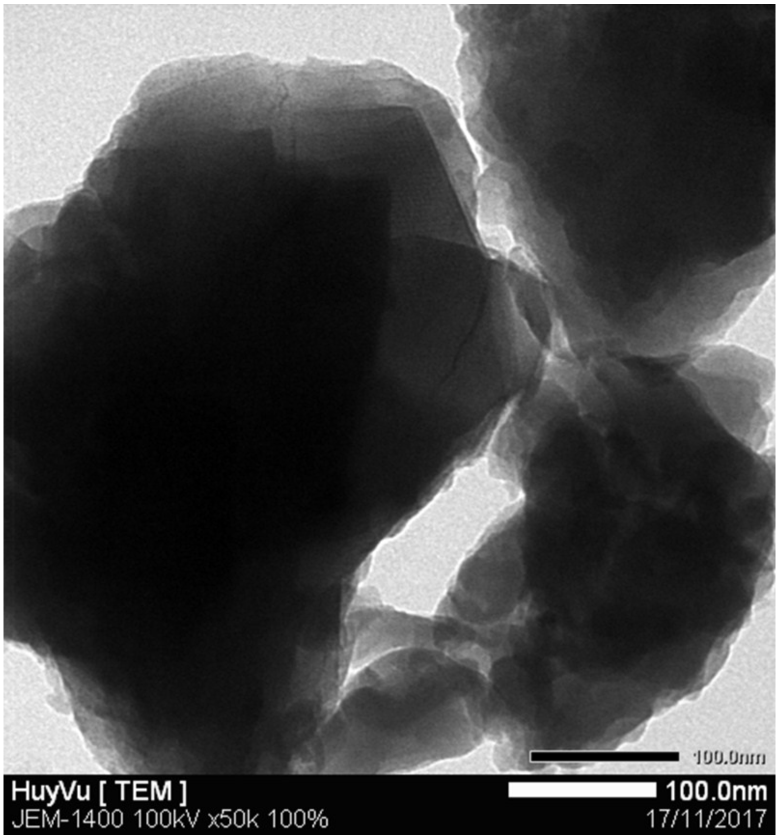

Fig. S3. TEM micrograph of the VNU-20.

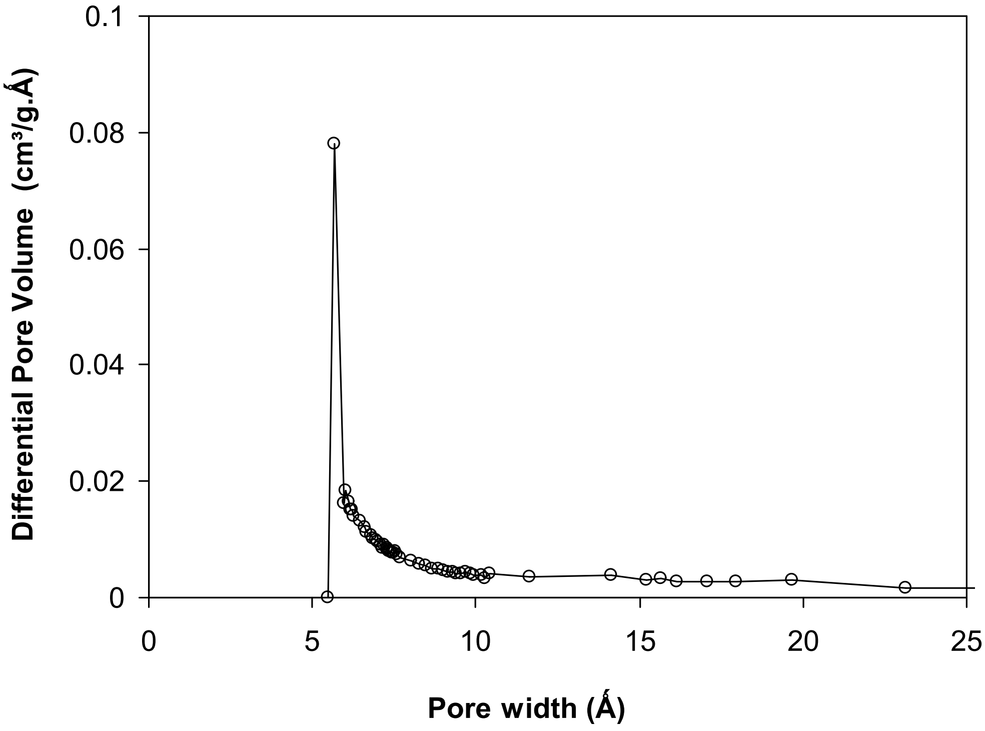

Fig. S4. Pore size distribution of the VNU-20.

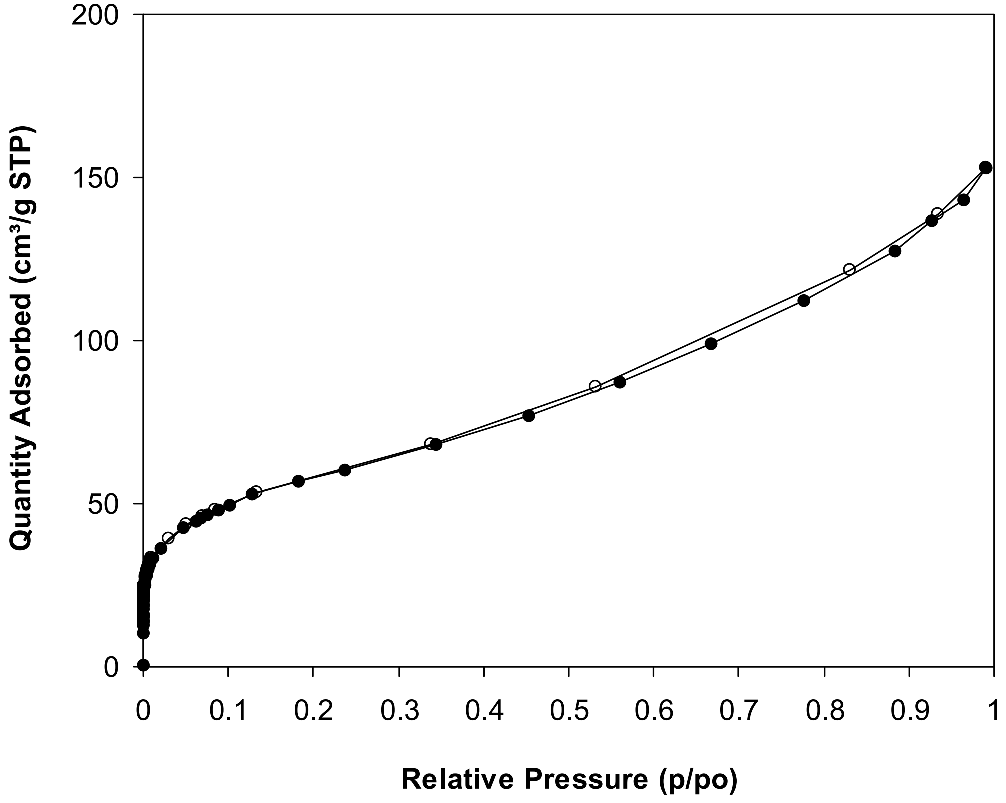

Fig. S5. Nitrogen adsorption/desorption isotherm of the VNU-20. Adsorption data are shown as closed circles and desorption data as open circles.

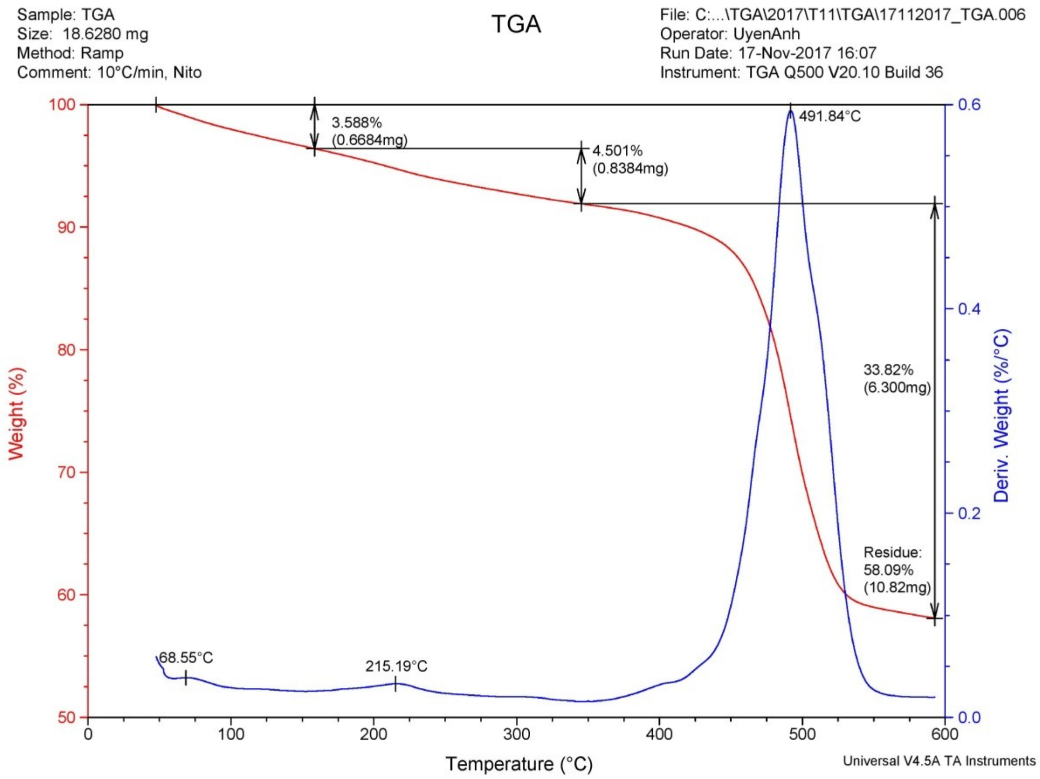

Fig. S6. TGA analysis of the VNU-20.

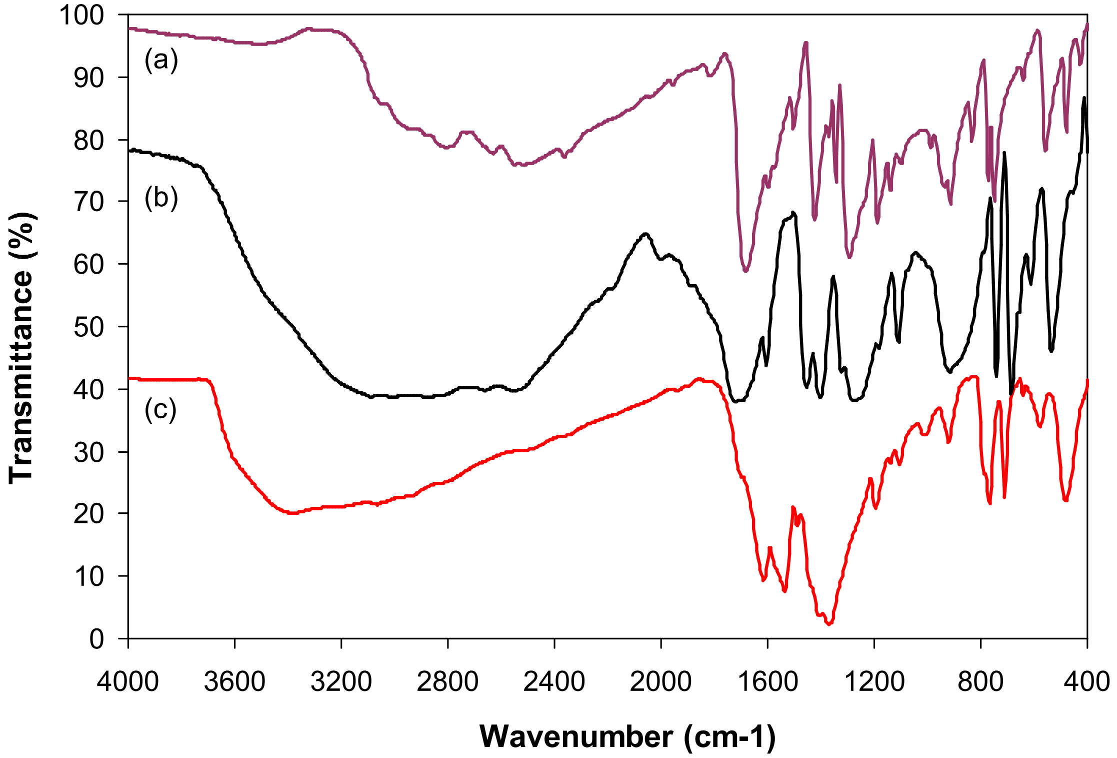

Fig. S7. FT-IR spectra of biphenyl-4,4’-dicarboxylic acid (a), 1,3,5-benzenetricarboxylic acid (b), and the VNU-20 (c).

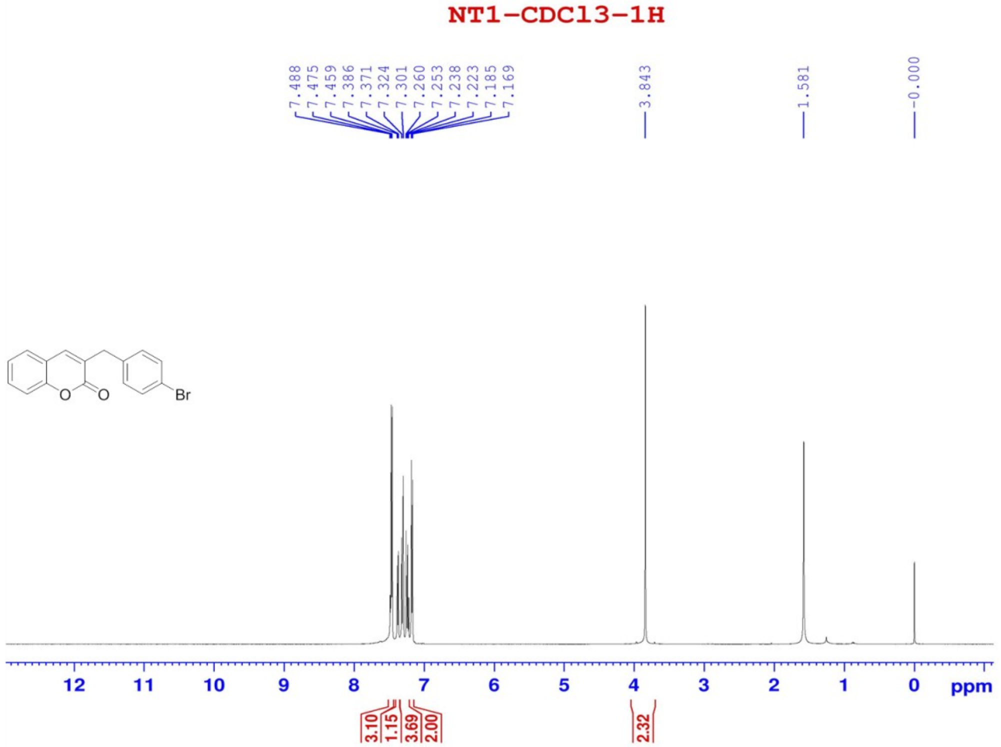

Fig.S8. ^{1}H-NMR spectra of 3-(4-bromobenzyl)-2H-chromen-2-one.

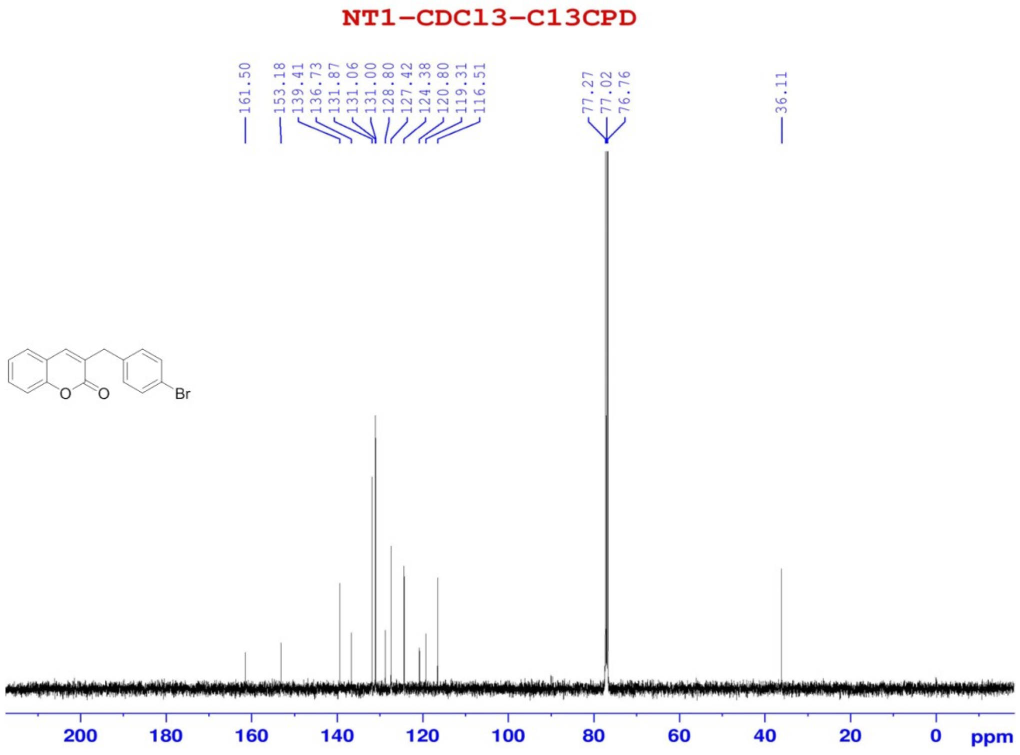

Fig.S9. ^{13}C-NMR spectra of 3-(4-bromobenzyl)-2H-chromen-2-one.

Characterization data for3-(4-bromobenzyl)-2H-chromen-2-one

Prepared as shown in the general experimental procedure and purified on silica gel (230-400 mesh or 37-63 µm, EtOAc/petroleum ether = 1:5 (v./v.), TLC silica gel 60 F_{254}, R_f = 0.5):White crystal, 72 % yield. ^{1}H-NMR (500 MHz, $CDCl_3$) 3.84 (s, 2H), 7.17 (d, *J = 8.0 Hz,* 2H), 7.22–7.26 (m, 1H), 7.31 (d, *J = 6.5 Hz,* 2H), 7.38 (d, *J = 8.0 Hz,* 1H), 7.45–7.48 (m, 3H). ^{13}C NMR ($CDCl_3$, 125 MHz) δ(ppm) 36.1, 116.5, 119.3, 120.8, 124.3, 127.4, 128.8, 131.0, 131.0, 131.8, 136.7, 139.4, 153.1, 161.5.

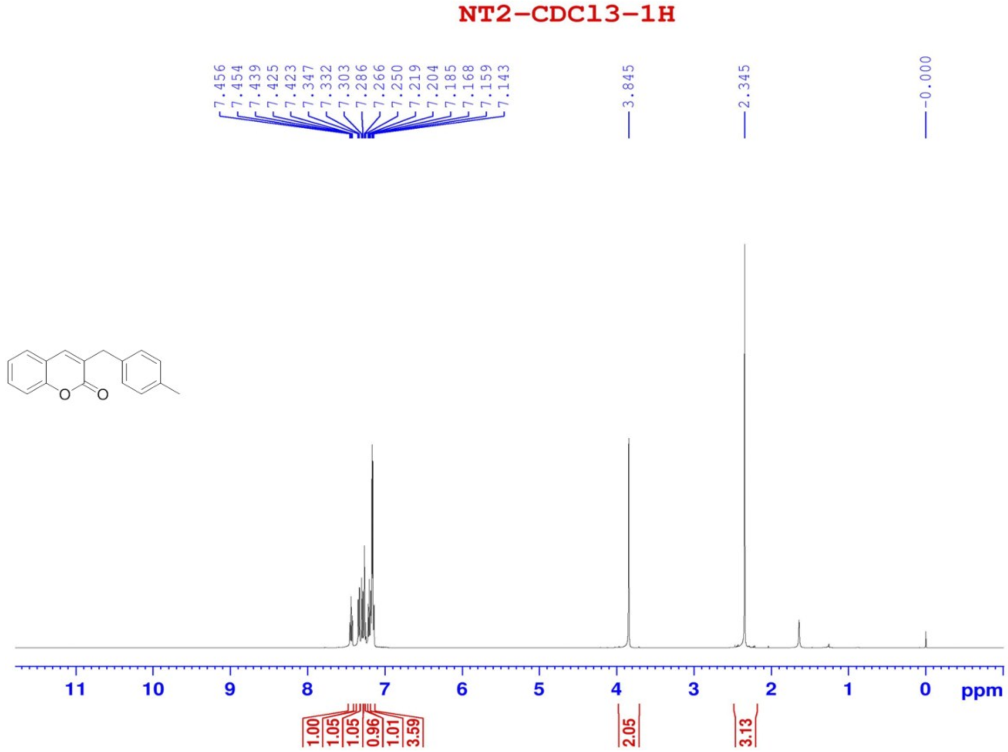

Fig.S10. ^{1}H-NMR spectra of 3-(4-methylbenzyl)-2H-chromen-2-one.

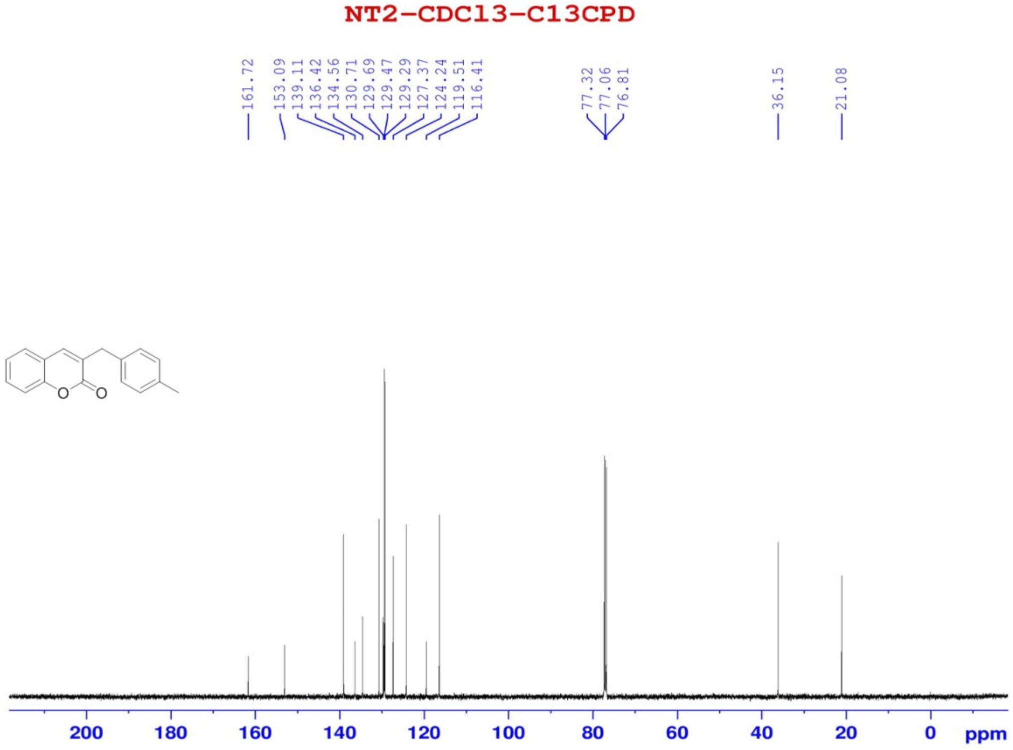

Fig.S11. ^{13}C-NMR spectra of 3-(4-methylbenzyl)-2H-chromen-2-one.

Characterization data for 3-(4-methylbenzyl)-2H-chromen-2-one

Prepared as shown in the general experimental procedure and purified on silica gel (230-400 mesh or 37-63 μm, EtOAc/petroleum ether = 1:5 (v./v.), TLC silica gel 60 F_{254}, R_f = 0.4): White crystal, 76 % yield. ^{1}H-NMR (500 MHz, $CDCl_3$) 2.34 (s, 3H), 3.84 (s, 2H), 7.14–7.26 (m, 5H), 7.28–7.34 (m, 3H), 7.42 (td, $J_1 = 8.5$ *Hz*, $J_2 = 1.0$ *Hz*, 1H). ^{13}C NMR ($CDCl_3$, 125 MHz) δ(ppm) 21.0, 36.1, 116.4, 119.5, 124.2, 127.3, 129.2, 129.4, 129.6, 130.7, 134.5, 136.4, 139.1, 153.0, 161.7.

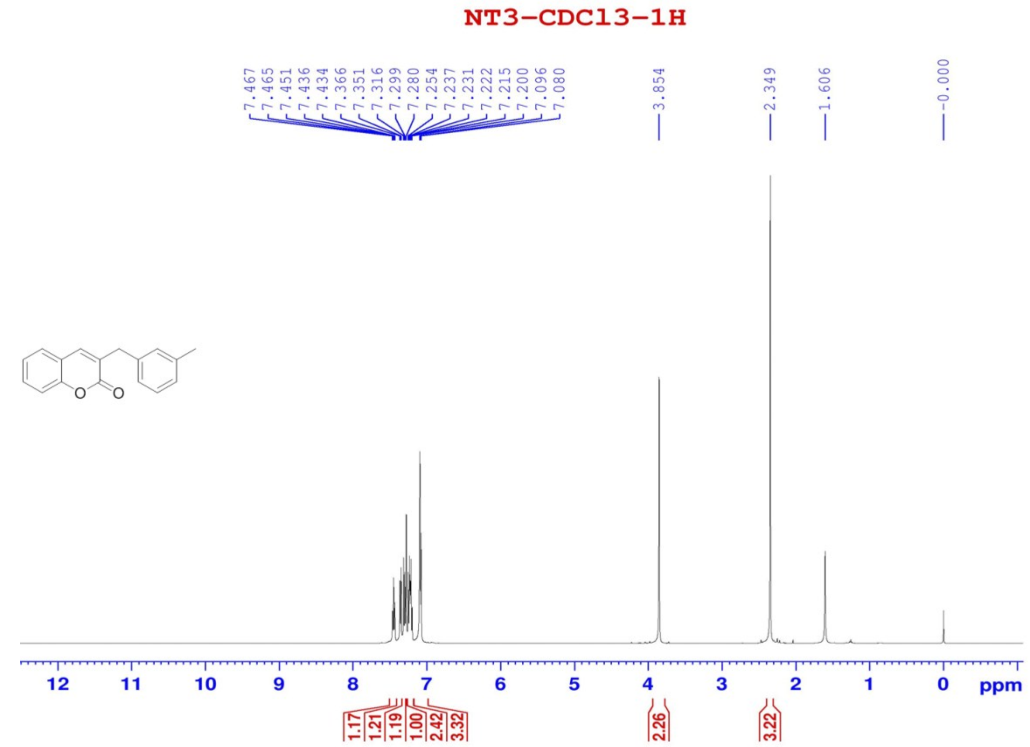

Fig.S12. ^{1}H-NMR spectra of 3-(3-methylbenzyl)-2H-chromen-2-one.

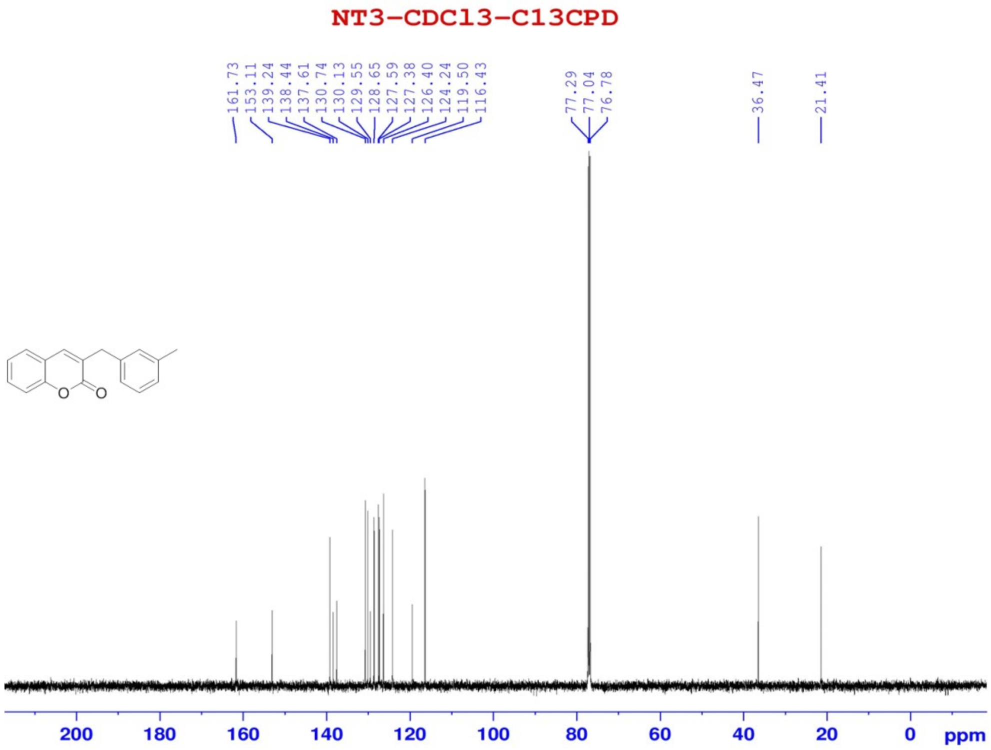

Fig.S13. ^{13}C-NMR spectra of 3-(3-methylbenzyl)-2H-chromen-2-one.

Characterization data for 3-(3-methylbenzyl)-2H-chromen-2-one

Prepared as shown in the general experimental procedure and purified on silica gel (230-400 mesh or 37-63 μm, EtOAc/petroleum ether = 1:5 (v./v.), TLC silica gel 60 F_{254}, R_f = 0.45): White crystal, 80 % yield. ^{1}H-NMR (500 MHz, $CDCl_3$) 2.34 (s, 3H), 3.85 (s, 2H), 7.08 (d, J = *8.0 Hz,* 3H), 7.20 – 7.31 (m, 4H), 7.35 (d, J = *7.5 Hz*, 1H), 7.45 (td, J_1 = *7.5 Hz,* J_2 = *1.0 Hz*, 1H). ^{13}C NMR ($CDCl_3$, 125 MHz) δ(ppm) 21.4, 36.4, 116.4, 119.5, 124.2, 126.4, 127.3, 127.5, 128.6, 129.5, 130.1, 130.7, 137.6, 138.4, 139.2, 153.1, 161.7.

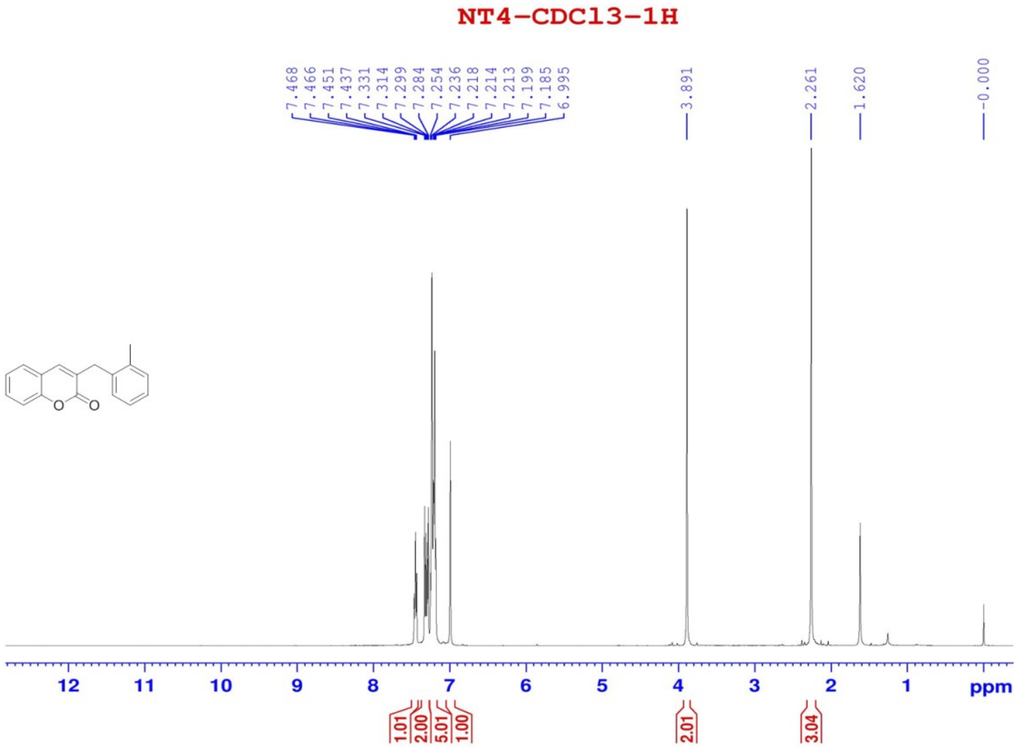

Fig.S14. ^{1}H-NMR spectra of 3-(2-methylbenzyl)-2H-chromen-2-one.

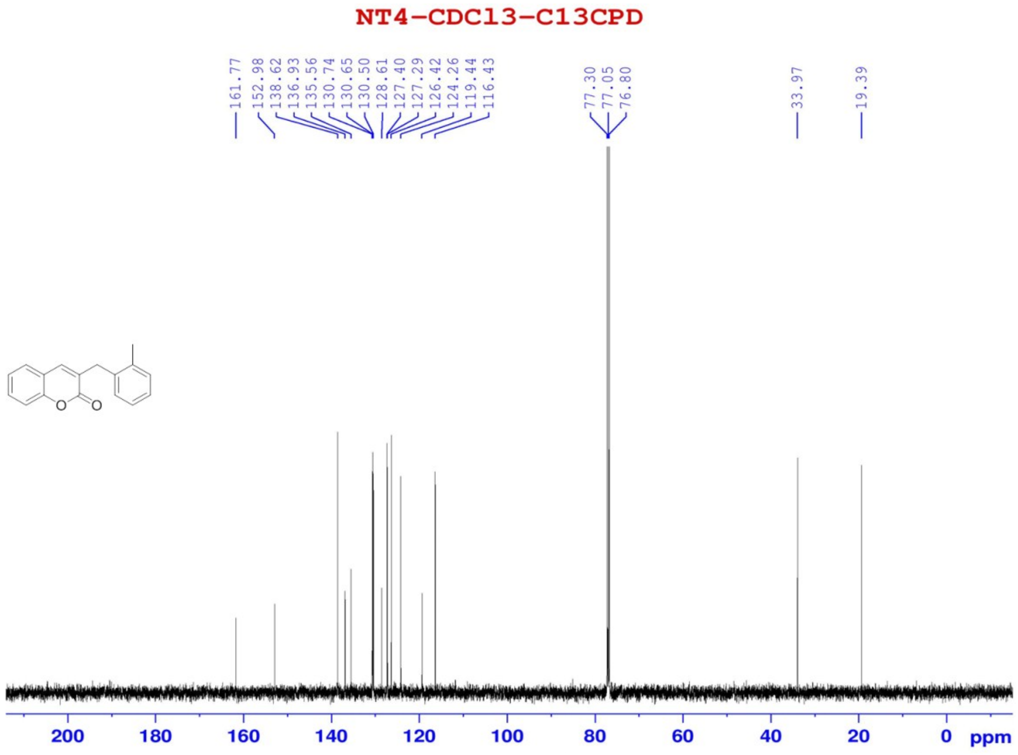

Fig.S15. ^{13}C-NMR spectra of 3-(3-methylbenzyl)-2H-chromen-2-one.

Characterization data for 3-(2-methylbenzyl)-2H-chromen-2-one

Prepared as shown in the general experimental procedure and purified on silica gel (230-400 mesh or 37-63 μm, EtOAc/petroleum ether = 1:5 (v./v.), TLC silica gel 60 F_{254}, R_f = 0.45): White crystal, 67 % yield. ^{1}H-NMR (500 MHz, $CDCl_3$) 2.26 (s, 3H), 3.89 (s, 2H), 6.99 (s, 1H), 7.18 – 7.25 (m, 5H), 7.28 – 7.33 (m, 2H), 7.43 (dt, $J_1 = 8.0$ *Hz,* $J_2 = 1.0$ *Hz*, 1H). ^{13}C NMR ($CDCl_3$, 125 MHz) δ(ppm) 19.3, 33.9, 116.4, 119.4, 124.2, 126.4, 127.2, 127.4, 128.6, 130.5, 130.6, 130.7, 135.5, 136.9, 138.6, 152.9, 161.7.

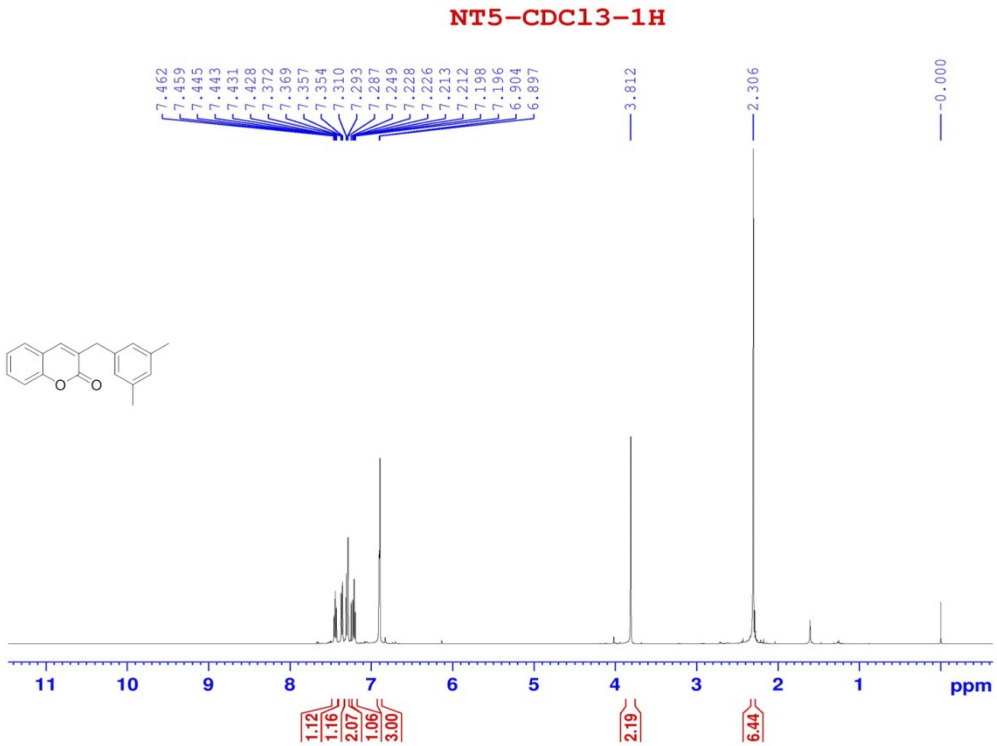

Fig.S16. ^{1}H-NMR spectra of 3-(3,5-dimethylbenzyl)-2H-chromen-2-one.

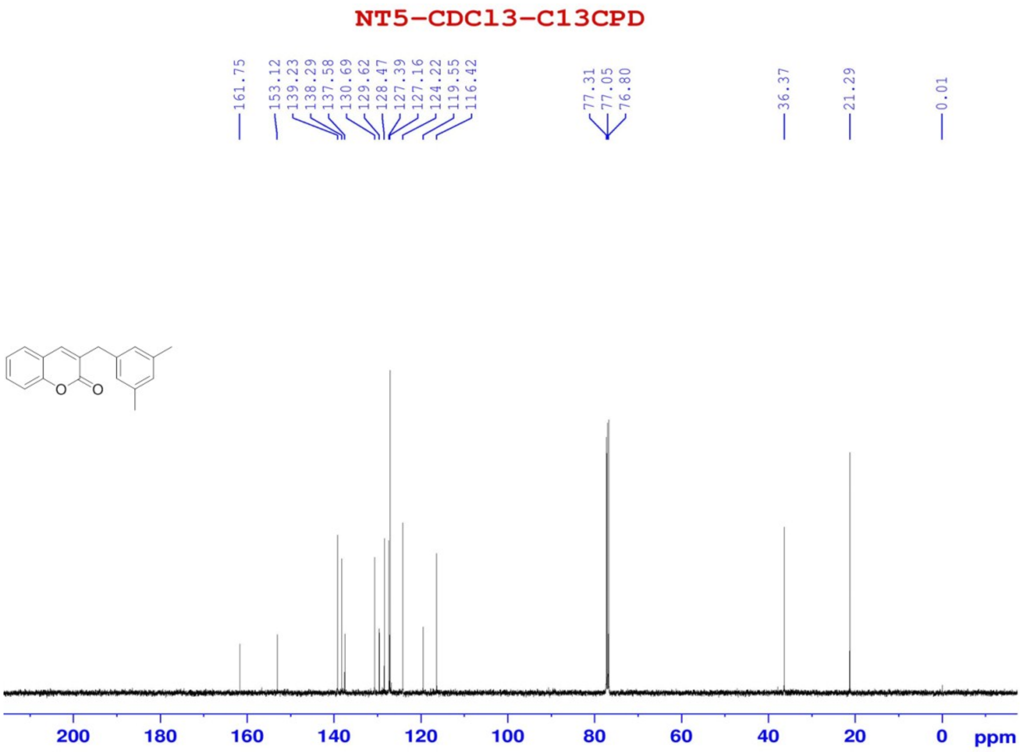

Fig.S17. ^{13}C-NMR spectra of 3-(3,5-dimethylbenzyl)-2H-chromen-2-one.

Characterization data for 3-(3,5-dimethylbenzyl)-2H-chromen-2-one

Prepared as shown in the general experimental procedure and purified on silica gel (230-400 mesh or 37-63 µm, EtOAc/petroleum ether = 1:5 (v./v.), TLC silica gel 60 F_{254}, R_f = 0.45): White crystal, 89 % yield. ^{1}H-NMR (500 MHz, $CDCl_3$) 2.30 (s, 6H), 3.81 (s, 2H), 6.89 (s, 3H), 7.21 (td, $J_1 = 7.5$ *Hz*, $J_2 = 1.0$ *Hz,* 1H), 7.28 – 7.31 (m, 2H), 7.35 (dd, $J_1 =$ *7.5 Hz*, $J_2 = 1.5$ *Hz,* 1H), 7.42 (td, $J_1 = 8.5$ *Hz*, $J_2 = 1.5$ *Hz*, 1H). ^{13}C NMR ($CDCl_3$, 125 MHz) δ(ppm) 21.9, 36.3, 116.4, 119.5, 124.2, 127.1, 127.3, 128.4, 129.6, 130.6, 137.5, 138.2, 139.2, 153.1, 161.7.

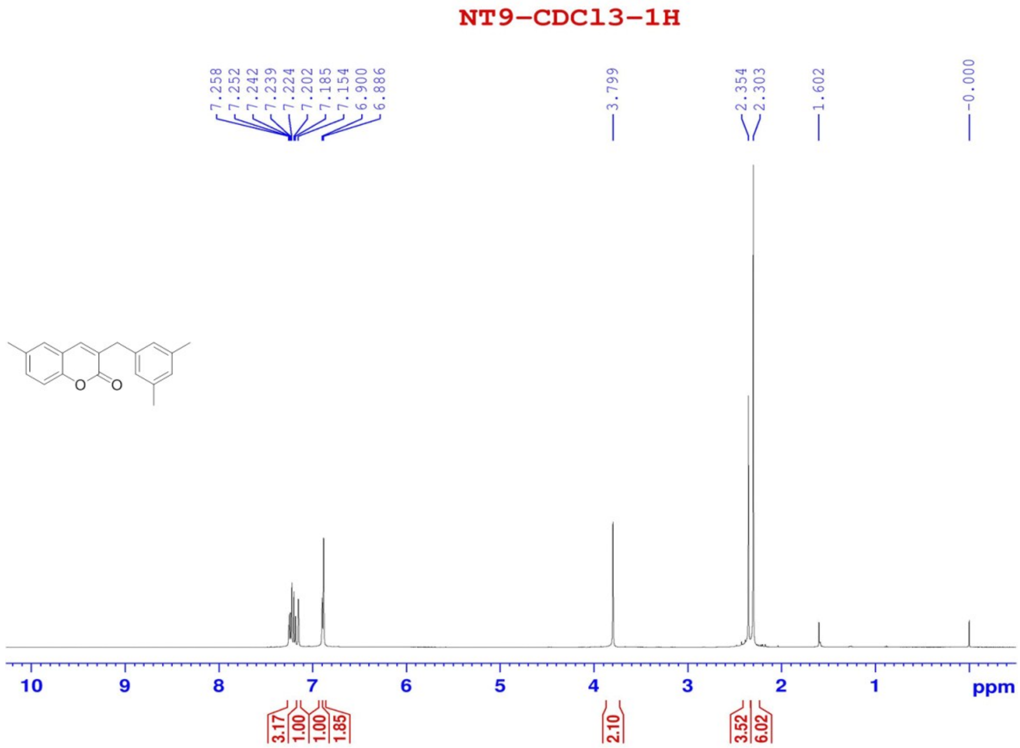

Fig.S18. ^{1}H-NMR spectra of 3-(3,5-dimethylbenzyl)-6-methyl-2H-chromen-2-one.

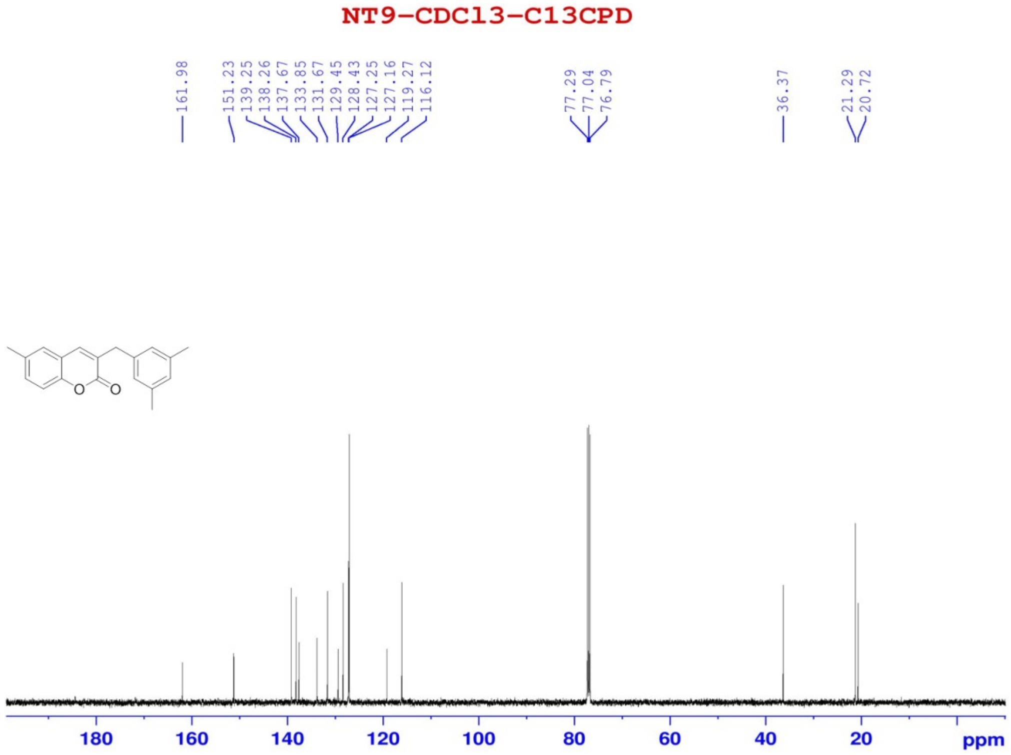

Fig.S19. ^{13}C-NMR spectra of 3-(3,5-dimethylbenzyl)-6-methyl-2H-chromen-2-one.

Characterization data for 3-(3,5-dimethylbenzyl)-6-methyl-2H-chromen-2-one

Prepared as shown in the general experimental procedure and purified on silica gel (230-400 mesh or 37-63 μm, EtOAc/petroleum ether = 1:5 (v./v.), TLC silica gel 60 F_{254}, R_f = 0.45): White crystal, 89 % yield. ^{1}H-NMR (500 MHz, $CDCl_3$) 2.30 (s, 6H), 2.35 (s, 3H), 3.79 (s, 2H), 6.88 (s, 2H),6.90 (s, 1H), 7.15 (s, 1H), 7.18-7.25 (m, 3H). ^{13}C NMR ($CDCl_3$, 125 MHz) δ(ppm) 20.7 , 21.2, 36.3, 116.1, 119.2, 127.1, 127.2, 128.4, 129.4, 131.6, 133.8, 137.6, 138.2, 139.2, 151.2, 161.9.

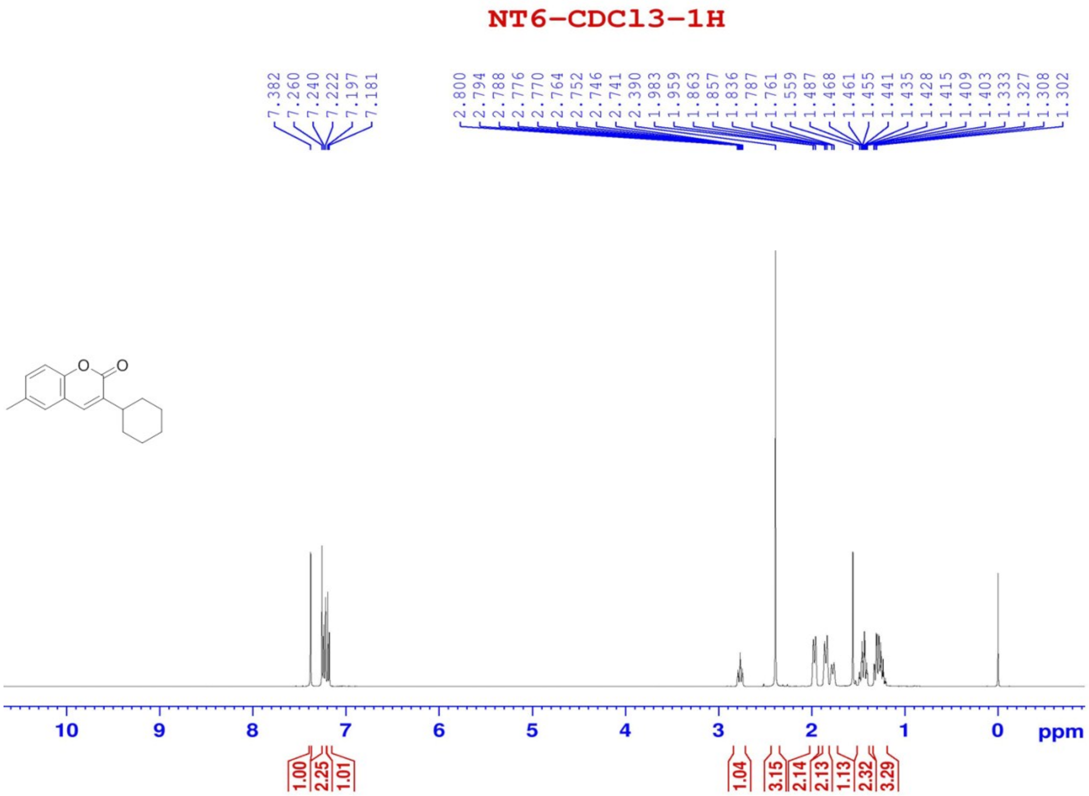

Fig.S20. ^{1}H-NMR spectra of 3-cyclohexyl-6-methyl-2H-chromen-2-one.

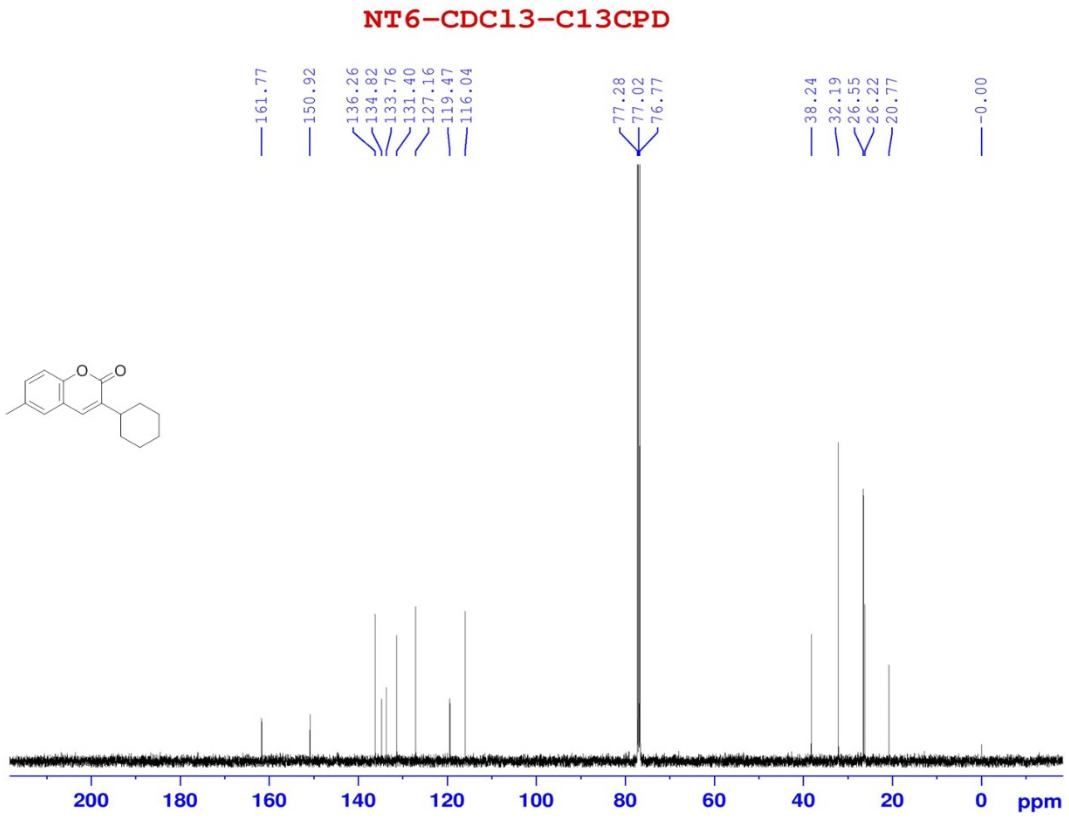

Fig.S21. ^{13}C-NMR spectra of 3-cyclohexyl-6-methyl-2H-chromen-2-one.

Characterization data for 3-cyclohexyl-7-methyl-2H-chromen-2-one

Prepared as shown in the general experimental procedure and purified on silica gel (230-400 mesh or 37-63 μm, EtOAc/petroleum ether = 1:15 (v./v.), TLC silica gel 60 F_{254}, R_f = 0.45): White solid, 60 % yield**.** ^{1}H NMR (500 MHz, $CDCl_3$) 1.22–1.33 (m, 3H), 1.40–1.48 (m, 2H), 1.77 (d, *J = 13.0 Hz*, 1H), 1.83 (dt, J_1 *= 13.5 Hz,* J_2 *= 3.0 Hz*, 2H), 1.96 (d, *J = 12.0 Hz*, 2H), 2.39 (s, 3H), 2.74 (tt, J_1 *= 12.0 Hz,* J_2 *= 2.5 Hz*, 1H), 7.18 (d, *J = 8.0 Hz*, 1H), 7.22 (d, *J = 8.0 Hz*, 1H), 7.26 (s, 1H), 7.38 (s, 1H) . ^{13}C NMR ($CDCl_3$, 125 MHz) δ(ppm) 20.7, 26.2, 26.5, 32.1, 38.2, 116.0, 119.4, 127.1, 131.4, 133.7, 134.8, 136.2, 150.9, 161.7.

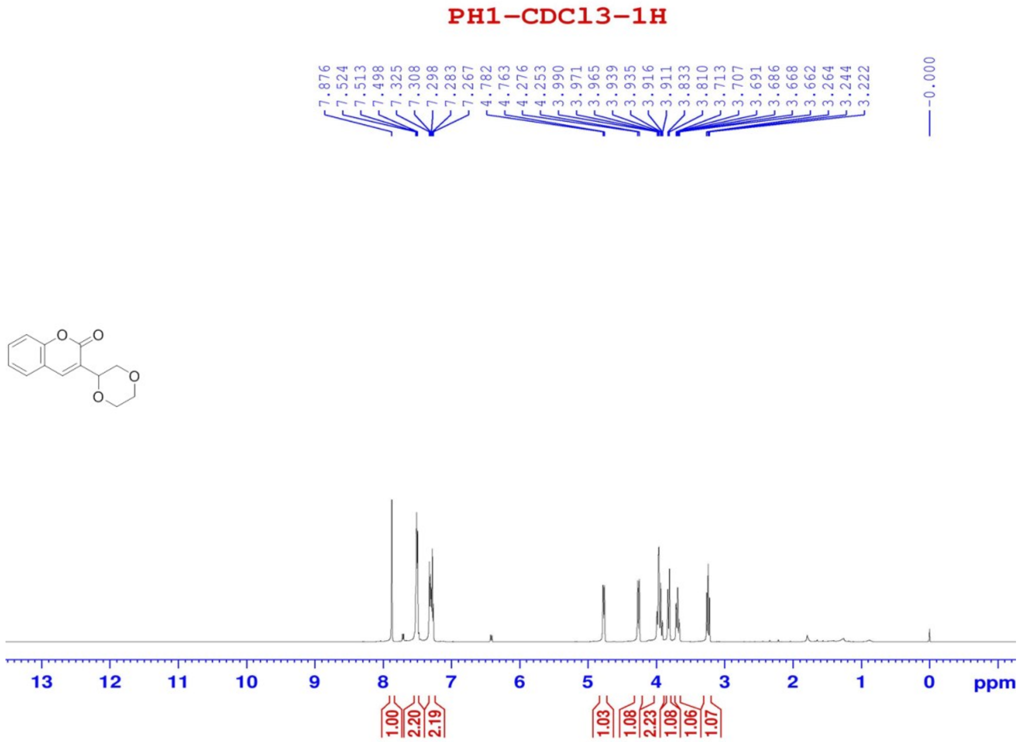

Fig.S22. ^{1}H-NMR spectra of 3-(1,4-dioxan-2-yl)-2H-chromen-2-one.

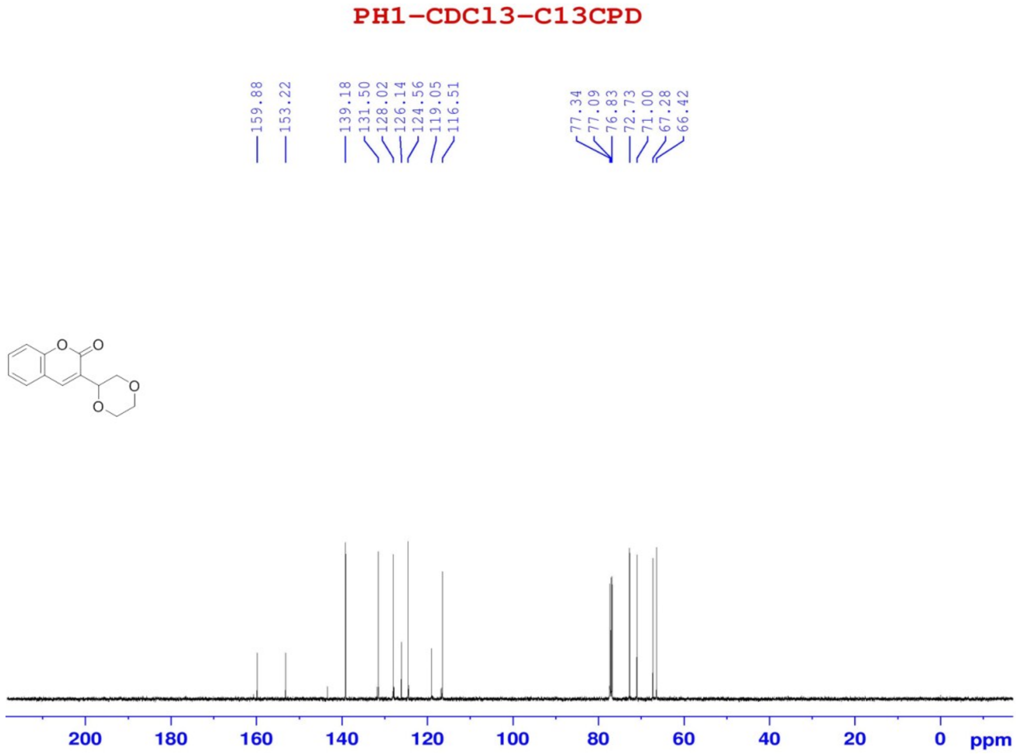

Fig.S23. ^{13}C-NMR spectra of 3-(1,4-dioxan-2-yl)-2H-chromen-2-one.

Characterization data for 3-(1,4-dioxan-2-yl)-2H-chromen-2-one

Prepared as shown in the general experimental procedure and purified on silica gel (230-400 mesh or 37-63 μm, EtOAc/petroleum ether = 1:15 (v./v.), TLC silica gel 60 F_{254}, R_f = 0.5): Colorless oil, 78 % yield **.** ^{1}H NMR (500 MHz, $CDCl_3$) 3.22 (t, *J = 11.0 Hz,* 1H), 3.66 (td, J_1 = *11.0 Hz,* J_2 = *2.5 Hz*, 1H), 3.81 (d, *J* = *11.5 Hz*, 1H), 3.91–3.99 (m, 2H), 4.25 (d, *J* = *11.5 Hz*, 1H), 4.76 (d, *J* = *9.5 Hz*, 1H), 7.26–7.32 (m, 2H), 7.49–4.52 (m, 2H), 7.87 (s, 1H) . ^{13}C NMR ($CDCl_3$, 125 MHz) δ(ppm) 66.4, 67.2, 71.0, 72.7, 116.5, 119.0, 124.5, 126.1, 128.0, 131.5, 139.1, 153.2, 159.8.

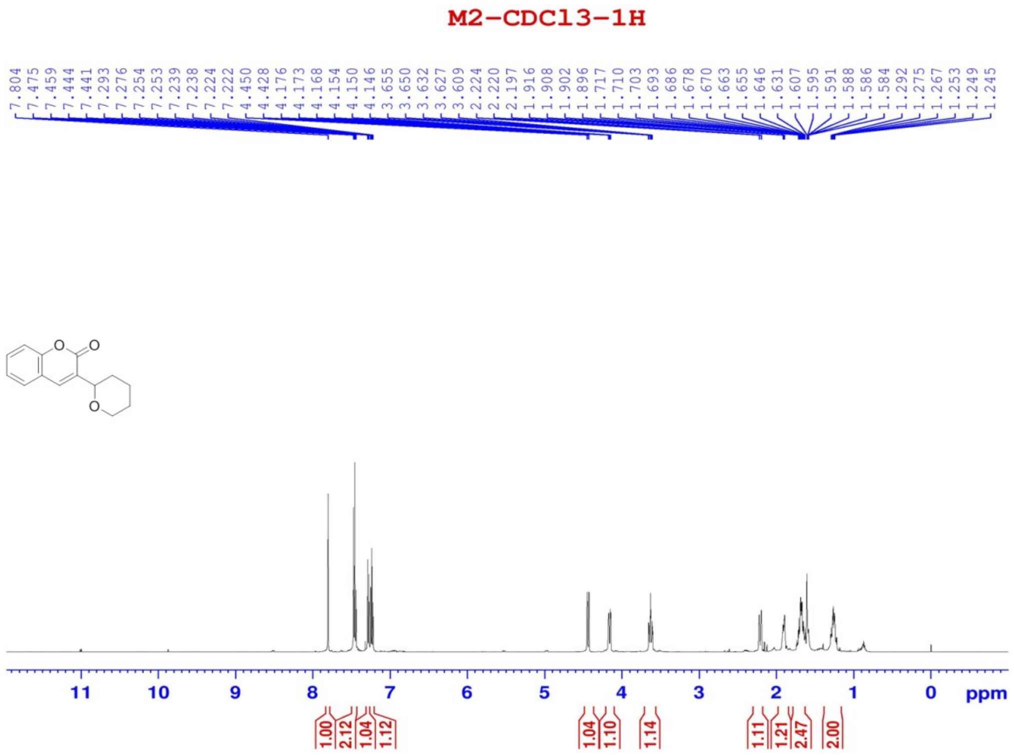

Fig.S24. ^{1}H-NMR spectra of 3-(tetrahydro-2H-pyran-2-yl)-2H-chromen-2-one.

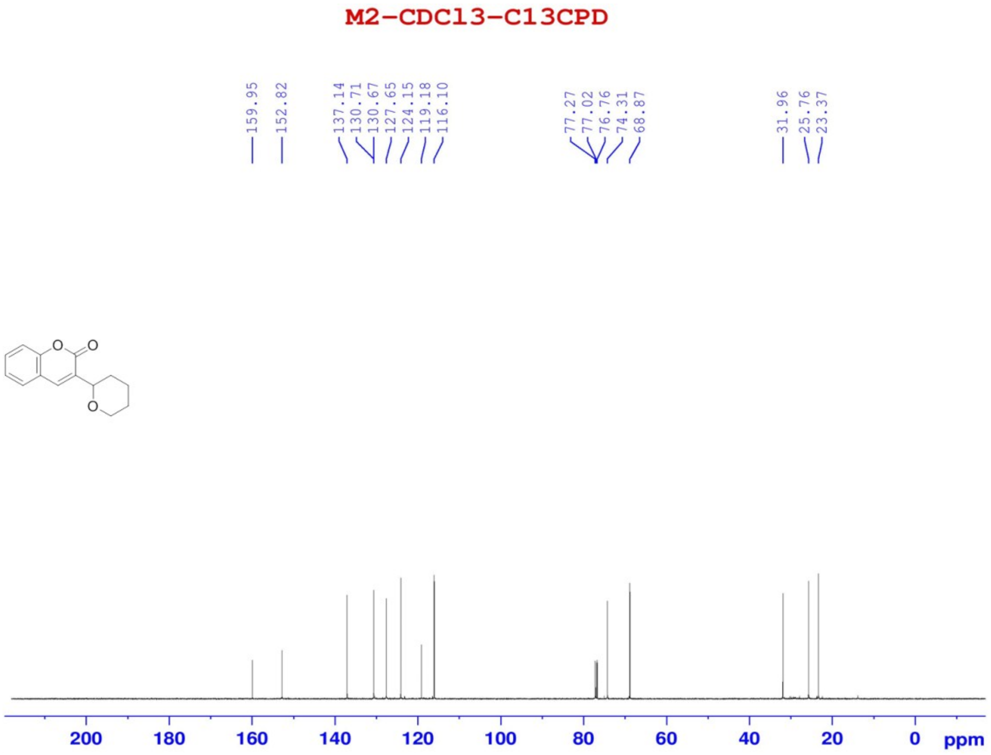

Fig.S25. ^{13}C-NMR spectra of 3-(tetrahydro-2H-pyran-2-yl)-2H-chromen-2-one.

Characterization data for 3-(tetrahydro-2H-pyran-2-yl)-2H-chromen-2-one

Prepared as shown in the general experimental procedure and purified on silica gel (230-400 mesh or 37-63 μm, EtOAc/petroleum ether = 1:15 (v./v.), TLC silica gel 60 F_{254}, R_f = 0.5): White solid, 74 % yield . ^{1}H NMR (500 MHz, $CDCl_3$) 1.24–1.30 (m, 2H), 1.63–1.71 (m, 2H), 1.89–1.92 (m, 1H), 2.19 (d, *J = 12.5 Hz,* 1H), 3.60 (td, J_1 = *11.5 Hz,* J_2 = *3.0 Hz*, 1H), 4.14 (dt, J_1 = *11.0 Hz,* J_2 = *2.0 Hz*, 1H), 4.42 (d, *J = 11.0 Hz,* 1H), 7.22 (td, J_1 = *7.5 Hz,* J_2 = *1.0 Hz*, 1H), 7.27 (d, *J = 8.5 Hz*, 1H), 7.44 –7.47(m, 2H), 7.80 (s, 1H). ^{13}C NMR ($CDCl_3$, 125 MHz) δ(ppm) 23.3, 25.7, 31.9, 68.8, 74.3, 116.1, 119.1, 124.1, 127.6, 130.6, 130.7, 137.1, 152.8, 159.9.

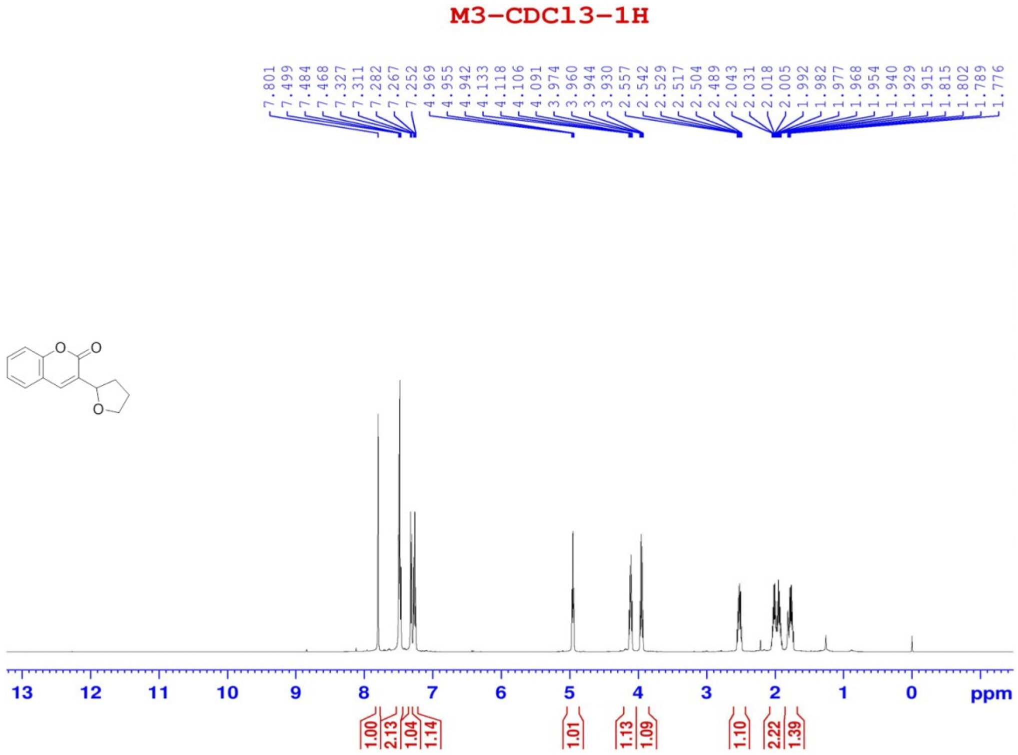

Fig.S26. ^{1}H-NMR spectra of 3-(tetrahydrofuran-2-yl)-2H-chromen-2-one.

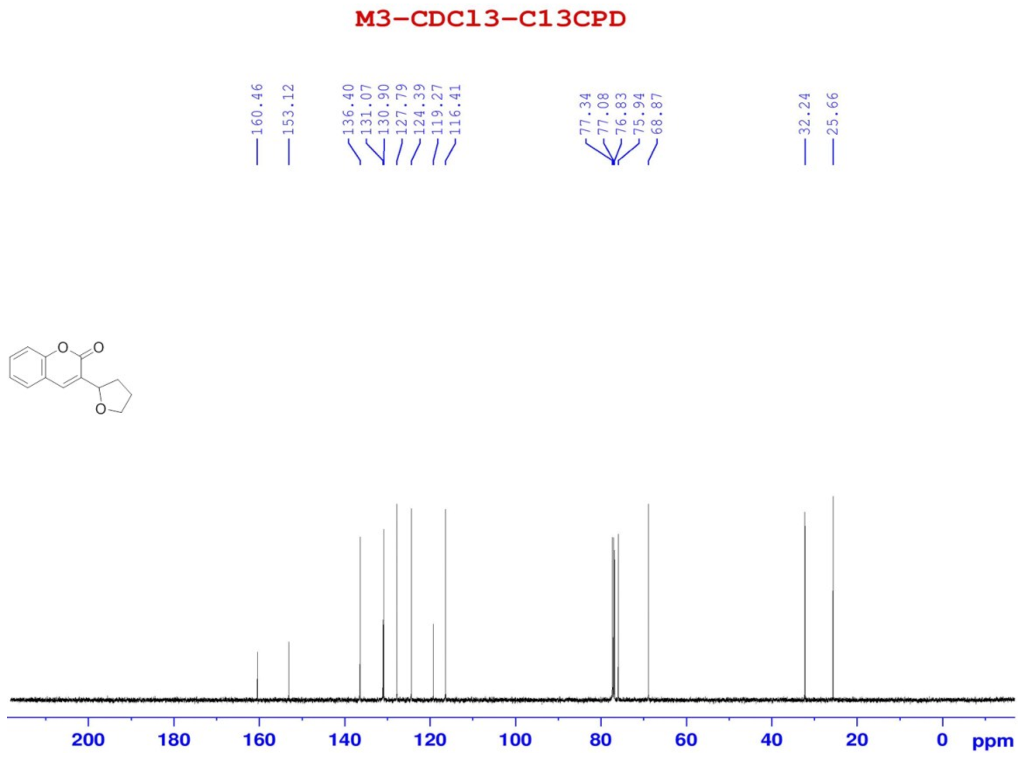

Fig.S27. ^{13}C-NMR spectra of 3-(tetrahydrofuran-2-yl)-2H-chromen-2-one.

Characterization data for 3-(tetrahydrofuran-2-yl)-2H-chromen-2-one

Prepared as shown in the general experimental procedure and purified on silica gel (230-400 mesh or 37-63 µm, EtOAc/petroleum ether = 1:15 (v./v.), TLC silica gel 60 F_{254}, R_f = 0.5): White solid, 73 % yield**.** ^{1}H NMR (500 MHz, $CDCl_3$) 1.73–1.81 (m, 1H), 1.91–2.04 (m, 2H), 2.48–2.55 (m*,* 1H), 3.93 (q, *J = 7.0 Hz*, 1H), 4.09 (q, *J = 7.0 Hz*, 1H), 4.94 (t, *J = 7.0 Hz*, 1H), 7.25 (t, *J = 7.5 Hz*, 1H), 7.31 (d, *J = 8.0 Hz*, 1H) 7.46–7.49 (m, 2H), 7.80 (s, 1H) . ^{13}C NMR ($CDCl_3$, 125 MHz) δ(ppm) 25.6, 32.2, 68.8, 75.9, 116.4, 119.2, 124.3, 127.7, 130.9, 131.0, 136.4, 153.1, 160.4.

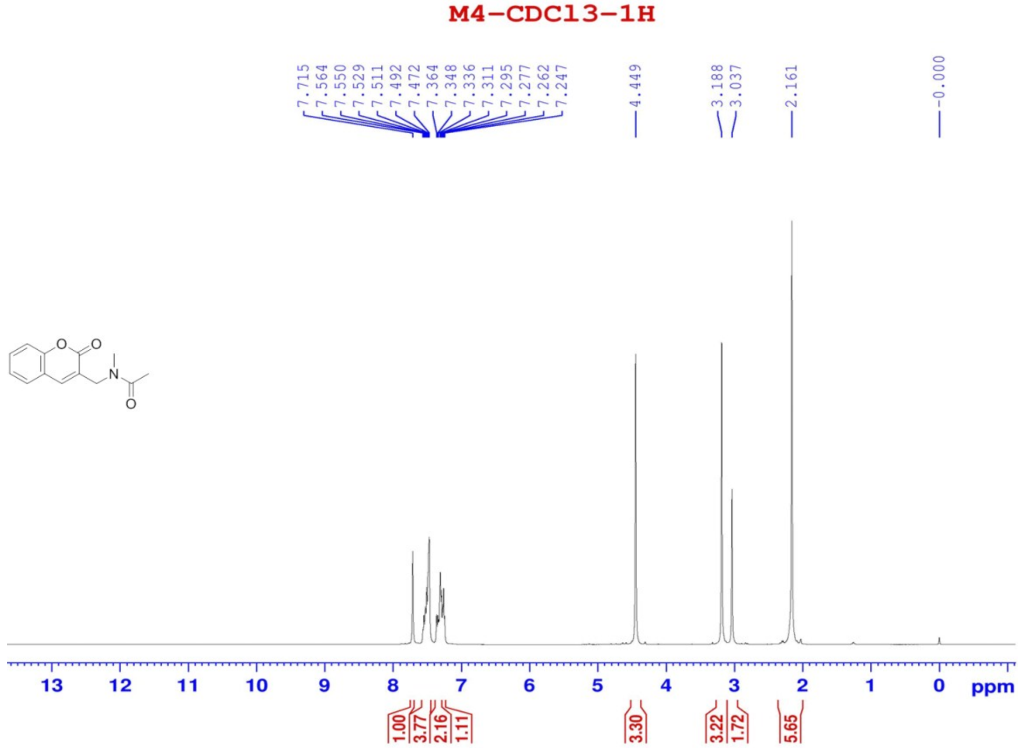

Fig.S28. ^{1}H-NMR spectra of N-methyl-N-((2-oxo-2H-chromen-3-yl)methyl)acetamide.

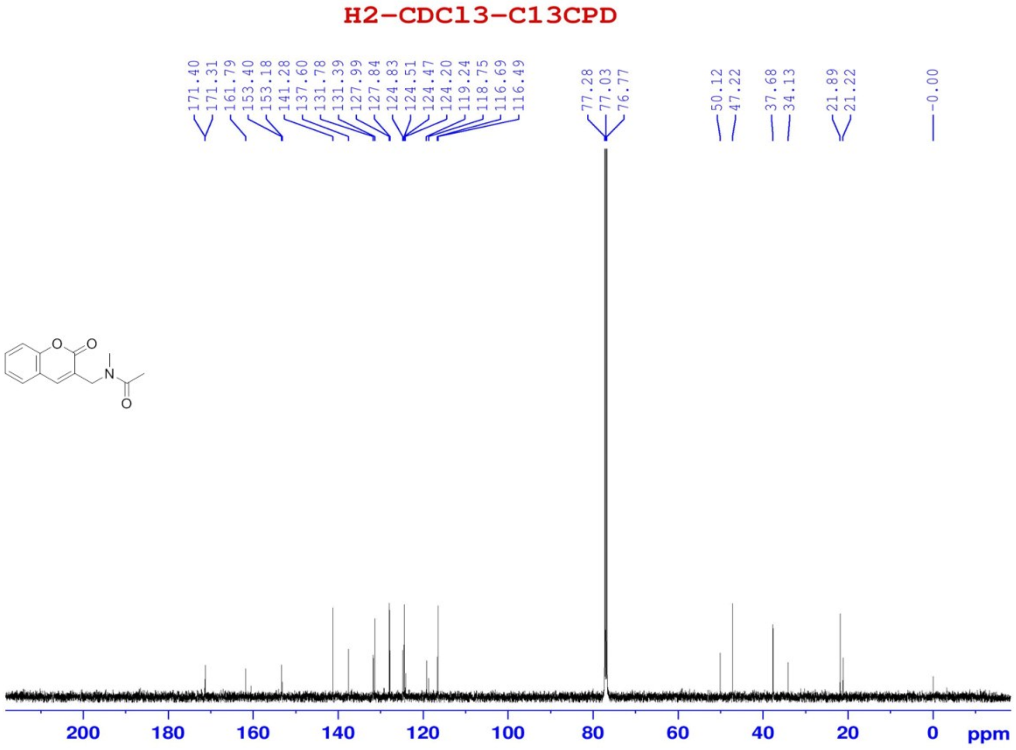

Fig.S29. ^{13}C-NMR spectra of N-methyl-N-((2-oxo-2H-chromen-3-yl)methyl)acetamide.

Characterization data for N-methyl-N-((2-oxo-2H-chromen-3-yl)methyl)acetamide

Prepared as shown in the general experimental procedure and purified on silica gel (230-400 mesh or 37-63 μm, ethyl acetate/hexane = 1:3 (v./v.), TLC silica gel 60 F_{254}, R_f = 0.75): Yellow solid, 97% yield . Observed as two rotamers of 65/35 ratio in ^{1}H-NMR. ^{1}H-NMR (500 MHz, $CDCl_3$) 2.16 (s, 3H), 3.19/3.04 (s, 3H), 4.45 (s, 2H), 7.25–7.36 (m, 2H), 7.47–7.56 (m, 2H), 7.72 (s, 1H). ^{13}C NMR ($CDCl_3$, 125 MHz) δ(ppm) 21.2, 21.8, 34.1, 37.7, 47.2, 50.1, 116.4, 116.6, 118.7, 119.2, 124.2, 124.4, 124.5, 124.8, 127.8, 127.9, 131.3, 131.7, 137.6, 141.2, 153.1, 153.4, 161.7, 171.3, 171.4.

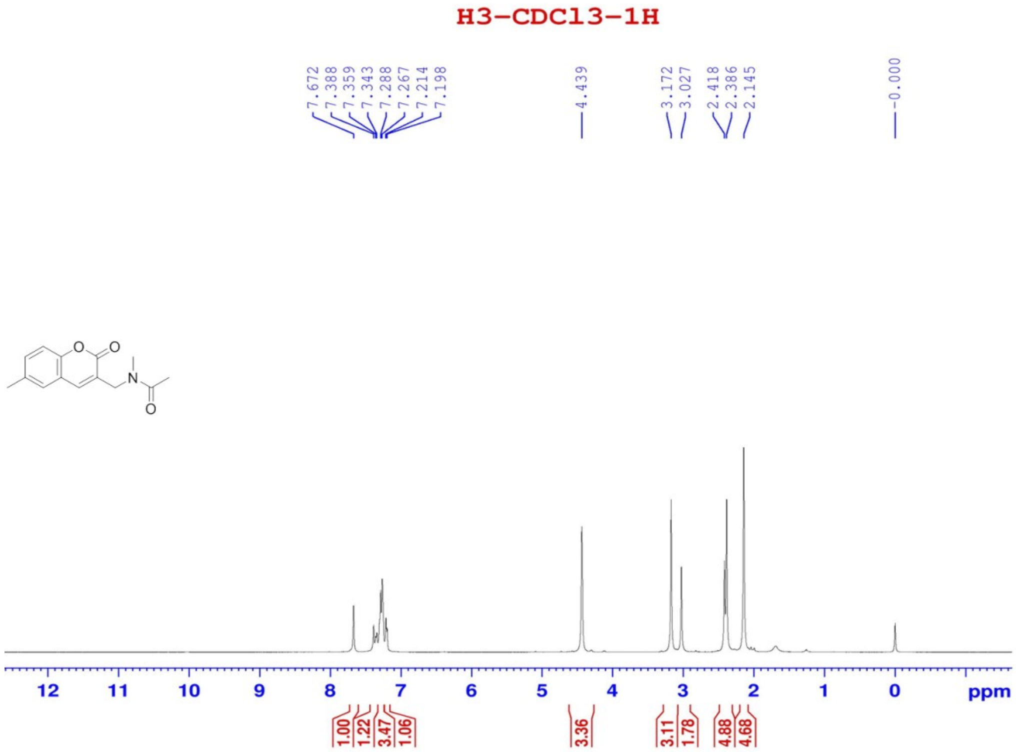

Fig.S30. ^{1}H-NMR spectra of N-methyl-N-((6-methyl-2-oxo-2H-chromen-3-yl)methyl)acetamide.

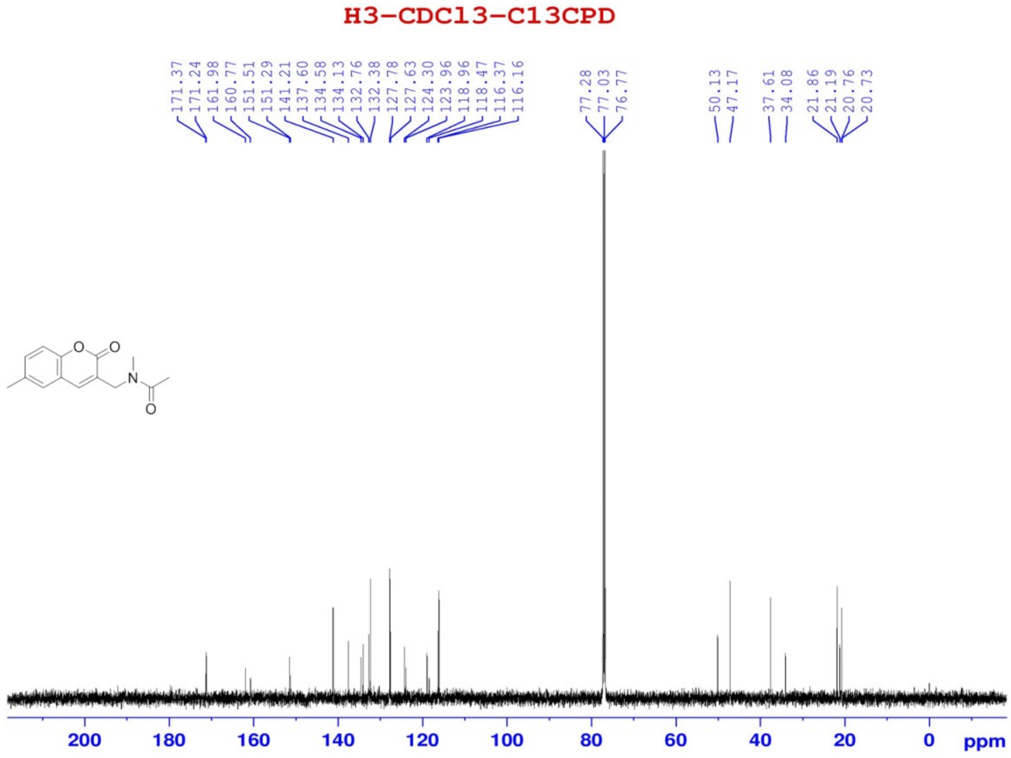

Fig.S31. ^{13}C-NMR spectra of N-methyl-N-((6-methyl-2-oxo-2H-chromen-3-yl)methyl)acetamide.

Characterization data for N-methyl-N-((6-methyl-2-oxo-2H-chromen-3-yl)methyl)acetamide

Prepared as shown in the general experimental procedure and purified on silica gel (230-400 mesh or 37-63 μm, ethyl acetate/hexane = 1:3 (v./v.), TLC silica gel 60 F_{254}, R_f = 0.75): White solid, 90% yield**.** Observed as two rotamers of 64/36 ratio in ^{1}H-NMR. ^{1}H-NMR (500 MHz, $CDCl_3$) 2.14 (s, 3H), 2.39/2.42 (s, 3H), 3.17/3.03 (s, 3H), 4.44 (s, 2H), 7.20–7.39 (m, 3H), 7.67 (s, 1H). ^{13}C NMR ($CDCl_3$, 125 MHz) δ(ppm) 20.7, 20.7, 21.1, 21.8, 34.0, 37.6, 47.1, 50.1, 116.1, 116.3, 118.4, 118.9, 123.9, 124.3, 127.6, 127.7, 132.3, 132.7, 134.1, 134.5, 137.6, 141.2, 151.2, 151.5, 160.7, 161.9, 171.2, 171.4.

GC yield determination

A series of solutions of 3-(3,5-dimethylbenzyl)-6-methyl-2*H*-chromen-2-one and diphenyl ether as internal standard with pre-determined concentrations were prepared. These solutions were analyzed by GC. The correlation between molar ratios vs peak area ratios of these solutions were plotted, giving a standard linear line. At different time intervals, reaction samples were analyzed by GC, and GC results provided the peak ratio between 3-(3,5-dimethylbenzyl)-6-methyl-2*H*-chromen-2-one and diphenyl ether and diphenyl ether. Based on the standard linear line, the molar ratio between 3-(3,5-dimethylbenzyl)-6-methyl-2*H*-chromen-2-one and diphenyl ether was then calculated, and susequently, the amount of 3-(3,5-dimethylbenzyl)-6-methyl-2*H*-chromen-2-one in the sample was determined. The GC yield was calculated based on the ratio between the amount of product in the sample over the theoretical amount product.

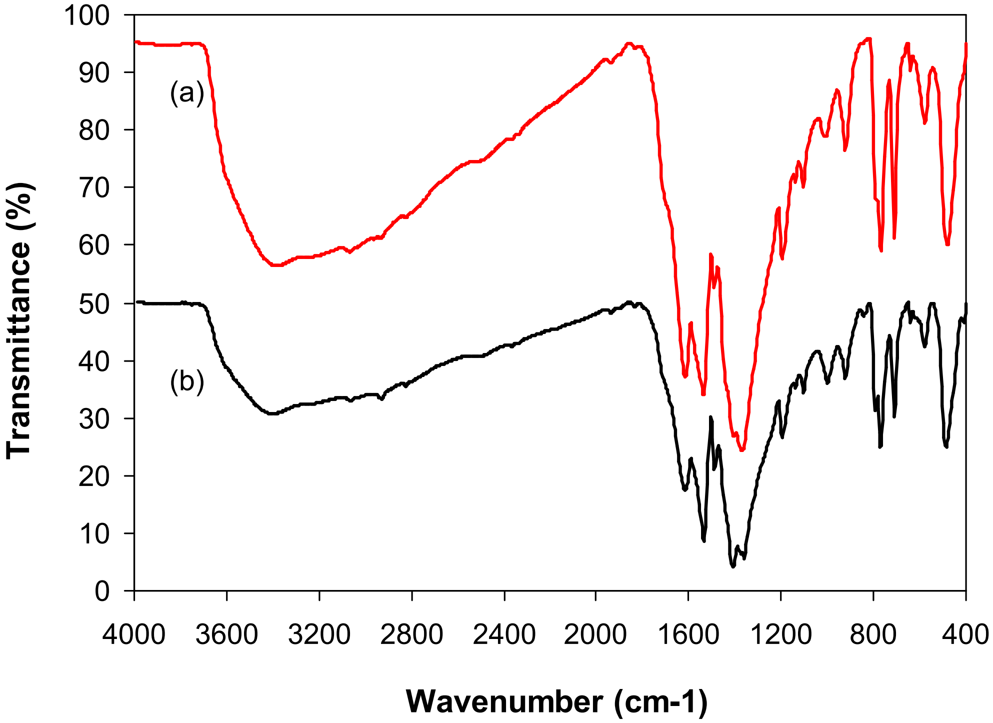

Fig. S32. FT-IR results of the fresh (a) and reutilized (b) VNU-20.

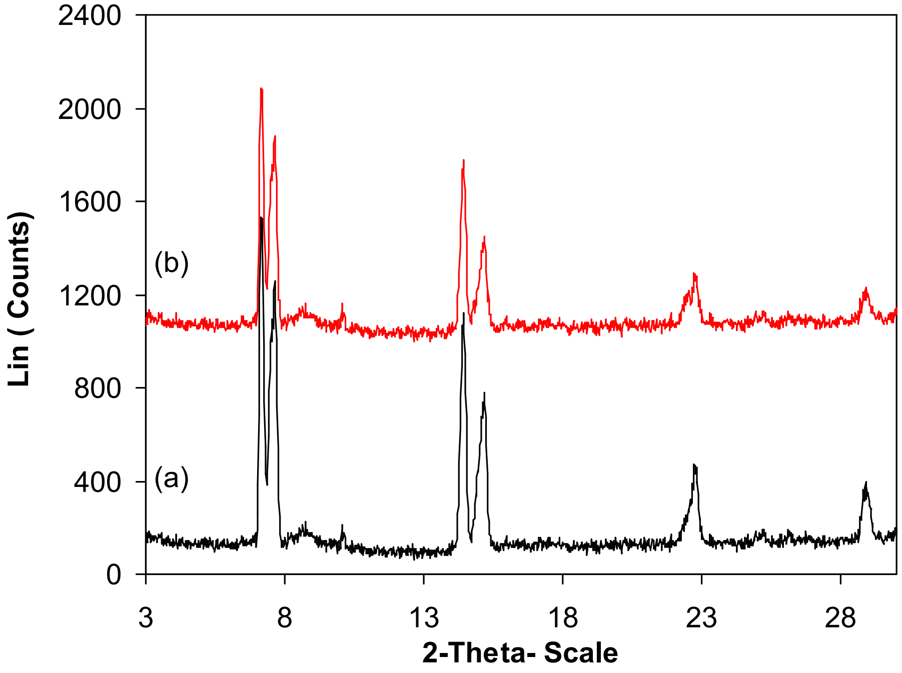

Fig. S33. XRD results of the fresh (a) and reutilized (b) VNU-20.

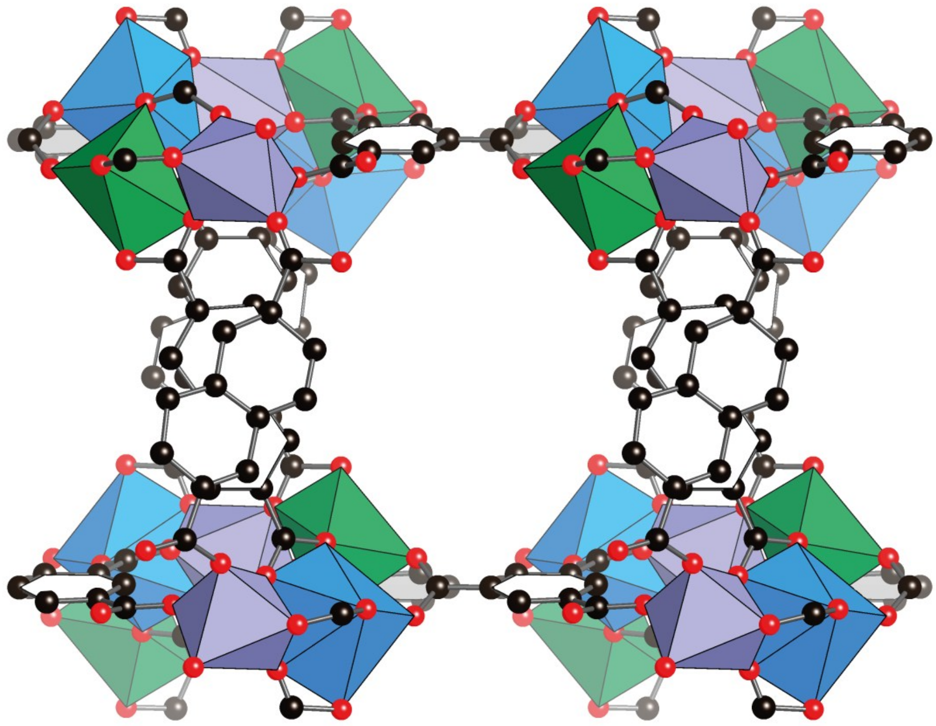

Fig. S34. The crystal structure of VNU-20 was presented in the super cell.

Synthesis of $Fe_3O(BDC)_3$

The $Fe_3O(BDC)_3$ was synthesized according to a slightly modified literature procedure [1]. In a typical preparation, a solid mixture of $FeCl_3.6H_2O$ (0.541 g, 2.0 mmol) and H_2BDC (H_2BDC=1,4-benzenedicarboxylic acid; 0.332 g, 2.0 mmol) was dissolved in a mixture of DMF (DMF = N,N'-dimethylformamide; 40 mL) and ethanol (40 mL). The resulting solution was distributed to eight 20-mL vials. The vials were tightly capped and then heated at 85 ^{o}C in an isothermal oven for 48 h, yielding light orange crystals. After cooling the vial to room temperature, the solid product was obtained by decanting with mother liquor and washed with DMF (3 x 10 mL) for 3 days. Solvent exchange was then performed with ethanol (3 x 10 mL) at room temperature for 3 days. The product was then dried under vacuum at 140 ^{o}C for 6 h.

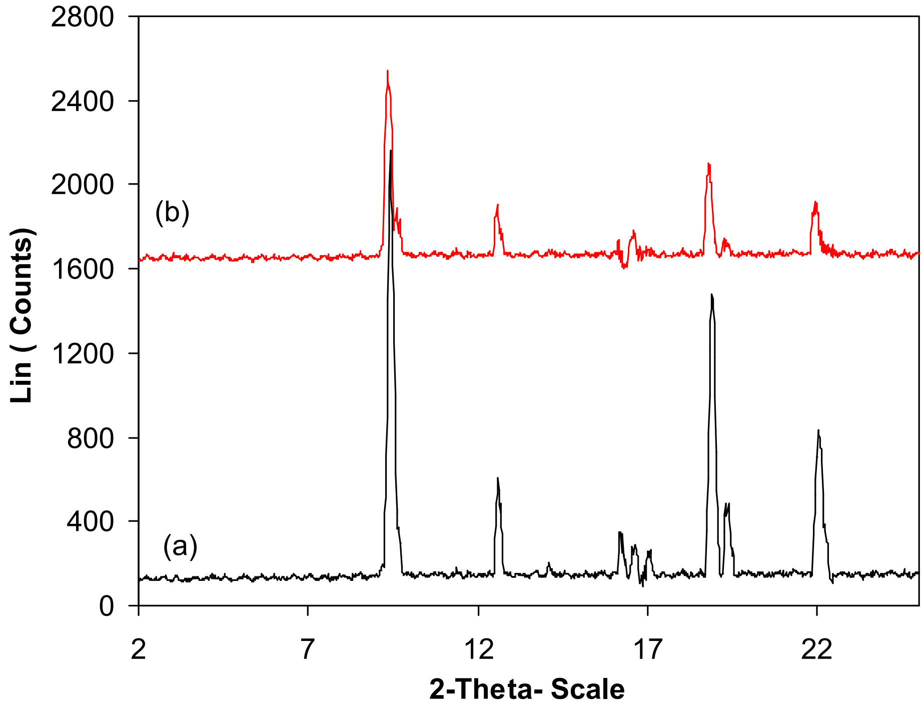

Fig. S35. XRD results of the fresh (a) and reutilized (b) $Fe_3O(BDC)_3$.

Synthesis of $Fe_3O(BPDC)_3$

The $Fe_3O(BPDC)_3$ was synthesized by solvothermal method using a literature procedure [2]. In a representative preparation, $FeCl_3.6H_2O$ (0.333 g, 1.24 mmol) in *N,N'*-dimethylformamide (20 ml) was added to the first beaker. A mixture of H_2BPDC (H_2BPDC = 4,4'-biphenyldicarboxylic acid; 0.16 g, 0.66 mmol) and acetic acid (CH_3COOH, 0.8 ml, 14 mmol) in *N,N'*-dimethylformamide (20 ml) was added to the second beaker. The former beaker was then poured into the latter along with *N,N'*-dimethylformamide (40 ml). The resulting mixture was vigorously stirred to achieve a clear solution. The solution was then equally added to pressurized vials. The vials were carefully capped and heated at 120 ^oC without interruption for 24 h. Orange crystals were produced on the wall of the vials throughout the time of the experiment. Subsequent to this period, the vials were cooled down to ambient temperature. The crystals were collected by decantation, and washed thoroughly with *N,N'*-dimethylformamide (3 x 20 ml). The solid was then immersed in dichloromethane (3 x 20 ml) at ambient temperature for solvent exchange. Afterwards, the product was dried at 160 ^oC under vacuum on a Shlenkline for 6 h.

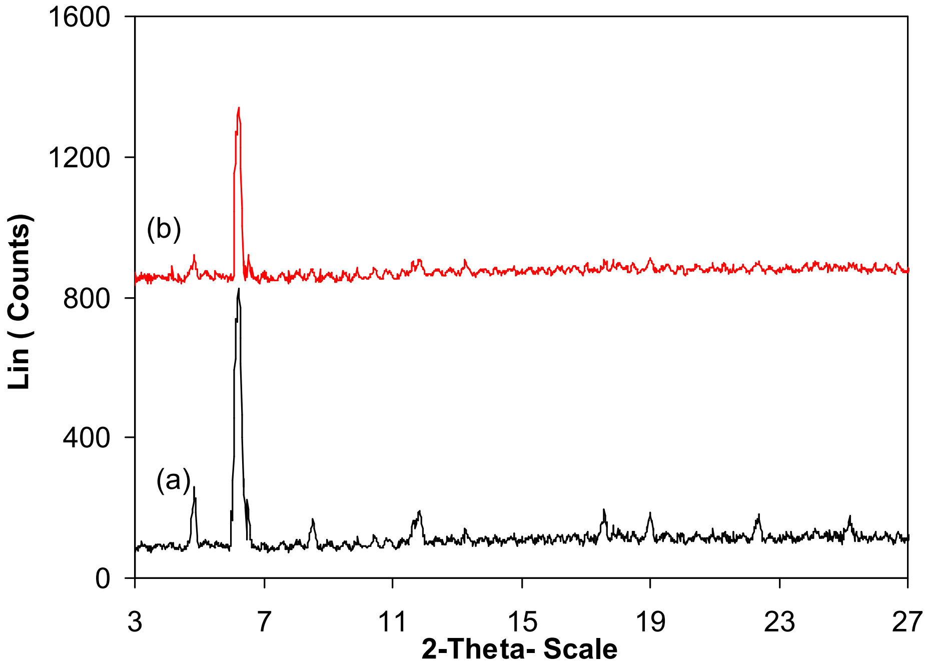

Fig. S36. XRD results of the fresh (a) and reutilized (b) $Fe_3O(BPDC)_3$.

Synthesis of $Cu_2(OBA)_2(BPY)$

The $Cu_2(OBA)_2(BPY)$ was synthesized by a literature approach [3]. In a typical procedure, 4,4′-oxybis(benzoic) acid (H_2OBA) (0.258 g, 1 mmol), copper (II) nitrate trihydrate ($Cu(NO_3)_2.3H_2O$) (0.242 g, 1 mmol), and 4,4′-bipyridine (BPY) (0.078 g, 0.5 mmol) were dissolved in the mixture of DMF and distilled water (14 mL, 11:3 v/v). The mixture was magnetically stirred for 30 min to obtain a clear solution. The solution was subsequently distributed to three 10 mL vials. The vials were carefully capped and heated at 85 ^oC in an isothermal oven for 48 h. Green crystals were formed during the experiment. After the vials were cooled to ambient temperature, the solid product in each vial was collected by decantation, and washed in DMF (3 x 10 mL). Solvent exchange was consequently performed with methanol (3 x 10 mL) at room temperature. The framework was then dried at 150 ^oC for 6 h under vacuum.

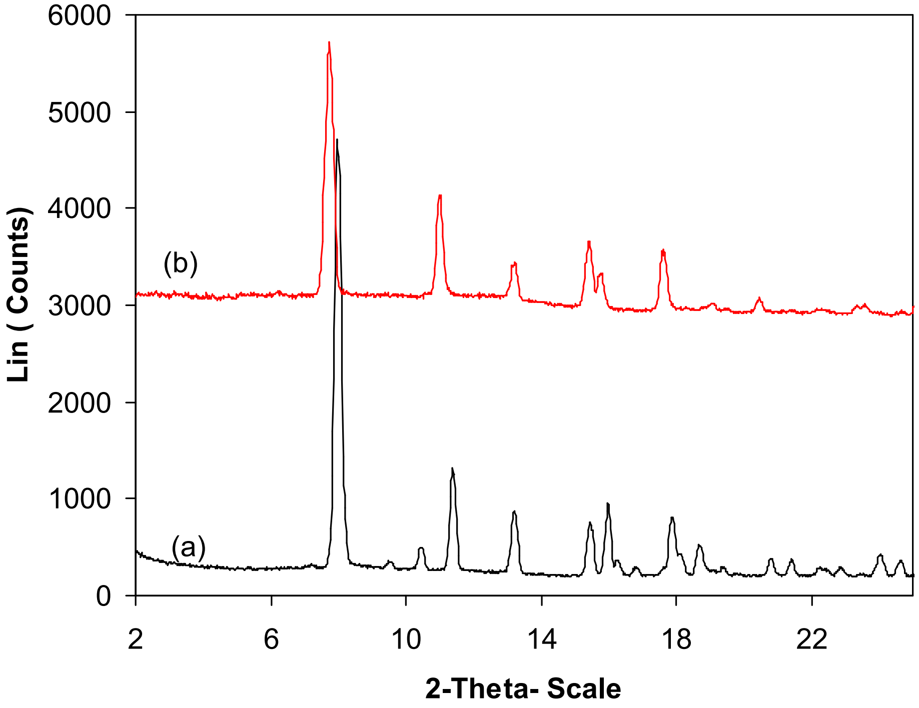

Fig. S37. XRD results of the fresh (a) and reutilized (b) $Cu_2(OBA)_2(BPY)$.

Synthesis of $Cu_2(BDC)_2(DABCO)$

The $Cu_2(BDC)_2(DABCO)$ was synthesized by a solvothermal method, according to a literature procedure [4]. a mixture of H_2BDC (H_2BDC = 1,4-benzenedicarboxylic acid; 0.506 g, 3.1 mmol), DABCO (DABCO = 1,4-diazabicyclo[2.2.2]octane; 0.188 g, 1.67 mmol), and $Cu(NO_3)_2 \cdot 3H_2O$ (0.8 g, 3.3 mmol) was dissolved in DMF (DMF = *N,N′*-dimethylformamide; 80 ml). The mixture was stirred for 2 h, and the resulting solution was then distributed to eight 10 ml vials. The vial was heated at 120 ^{o}C in an isothermal oven for 48 h, forming blue crystals. After cooling the vial to room temperature, the solid product was removed by decanting with mother liquor and washed with DMF (3 x 10 ml). Solvent exchange was carried out with methanol (3 x 10 ml) at room temperature. The product was then dried at 140 ^{o}C for 6 h under vacuum.

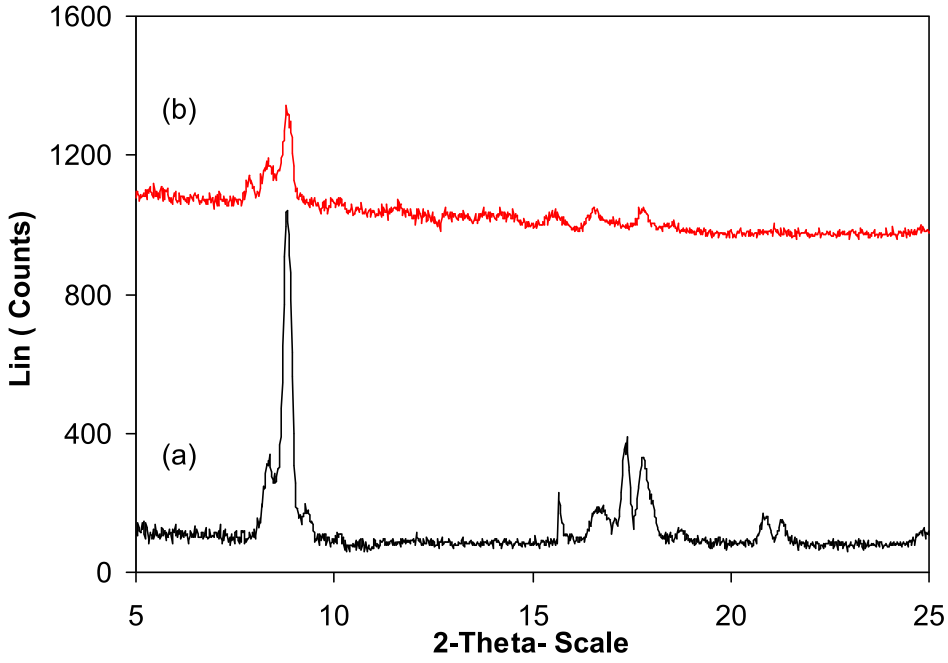

Fig. S38. XRD results of the fresh (a) and reutilized (b) $Cu_2(BDC)_2(DABCO)$.

Synthesis of $Cu_2(BPDC)_2(DABCO)$

The $Cu_2(BPDC)_2(DABCO)$ was synthesized according to a modified literature procedure [5]. In a typical procedure synthesis, a mixture of H_2BPDC (H_2BPDC = biphenyl-4,4'-dicarboxylic acid; 0.12 g, 0.50 mmol), DABCO (DABCO = 1,4-Diazabicyclo[2.2.2]octane; 0.03 g, 0.268 mmol), and $Cu(NO_3)_2 \cdot 3H_2O$ (0.12 g, 0.50 mmol) was dissolved in a mixture of DMF (DMF = N,N-dimethylformamide; 35 ml), and methanol (5 ml). The solution was magnetically stirred for 2 h before centrifuging. The resulting solution was then distributed into eight 20 ml vials. The vials were heated at 120 ^oC in an isothermal oven for 48 h. After cooling the vials to room temperature, the solid product in each vial was recovered by decanting with mother liquor and washed with DMF (3× 10 ml). Solvent exchange was then carried out with DCM (dichloromethane) (3× 10 ml) at room temperature. The product was then dried at 140 ^oC for 6 h under vacuum.

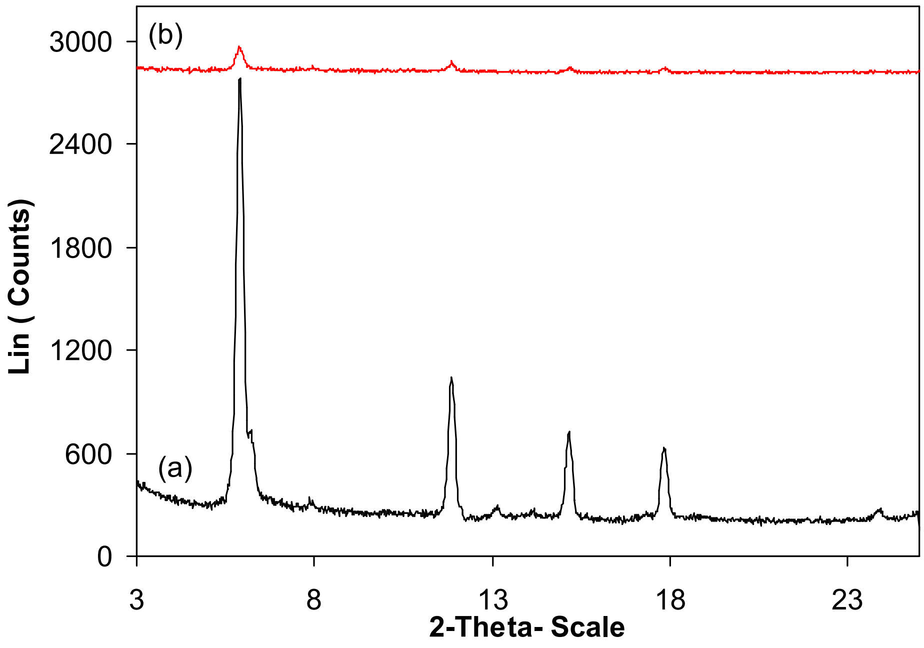

Fig. S39. XRD results of the fresh (a) and reutilized (b) $Cu_2(BPDC)_2(DABCO)$.

References

1. M. Anbia, V. Hoseini, S. Sheykhi, *J. Ind. Eng. Chem.* 2012, **18**, 1149-1152.
2. M. Dan-Hardi, H. Chevreau, T. Devic, P. Horcajada, G. Maurin, G. Férey, D. Popov, C. Riekel, S. Wuttke, J.-C. Lavalley, A. Vimont, T. Boudewijns, D.E.D. Vos, C. Serre, *Chem. Mater.* 2012, **24**, 2486-2492.
3. L. Tang, D. Li, F. Fu, Y. Wu, Y. Wang, H. Hu and E. Wang, *J. Mol. Struct.*, 2008, **888**, 344-353
4. K. Tan, N. Nijem, P. Canepa, Q. Gong, J. Li, T. Thonhauser, Y.J. Chabal, *Chem. Meter.* 2012, **24**, 3153-3167.
5. S. Seki, W. Mori, *J. Phys. Chem. B*, 2002, **106**, 1380-1385.
